# Augmented CD4^+^ T-cell and humoral responses after repeated annual influenza vaccination with the same vaccine component A/H1N1pdm09 over 5 years

**DOI:** 10.1038/s41541-018-0069-1

**Published:** 2018-08-14

**Authors:** Mai-Chi Trieu, Fan Zhou, Sarah Larteley Lartey, Saranya Sridhar, Siri Mjaaland, Rebecca Jane Cox

**Affiliations:** 1 The Influenza Centre, Department of Clinical Science, University of Bergen, Bergen, Norway; 2 K.G. Jebsen Centre for Influenza Vaccine Research, Department of Clinical Science, University of Bergen, Bergen, Norway; 3 Jenner Institute, University of Oxford, Oxford, UK; 4 Department of Infectious Disease Immunology, Norwegian Institute of Public Health, Oslo, Norway; 5 Department of Research and Development, Haukeland University Hospital, Bergen, Norway; 6 Present Address: Sanofi Pasteur, 1541 Avenue Marcel Mérieux, 69280 Marcy l’Etoile, France

## Abstract

Annual seasonal influenza vaccination is recommended for high-risk populations and often occupational groups such as healthcare workers (HCWs). Repeated annual vaccination has been reported to either have no impact or reduce antibody responses or protection. However, whether repeated vaccination influences T-cell responses has not been sufficiently studied, despite the increasing evidence of the protective roles of T-cell immunity. Here, we explored the impact of repeated annual vaccination with the same vaccine strain (H1N1pdm09) over multiple seasons in the post-2009 pandemic era and showed that repeated vaccination increased both T-cell and humoral responses. Using the T-cell FluroSpot and intracellular cytokine-staining, the hemagglutination inhibition (HI), and the memory B-cell (MBC) ELISpot assays, we investigated pre- and postvaccination T cells, antibodies, and MBCs in a cohort of HCWs repeatedly vaccinated with H1N1pdm09 for 5 years (pandemic vaccination in 2009 and subsequently annual seasonal vaccination containing H1N1pdm09 during 2010–2013). We found that the prevaccination H1N1pdm09-specific T cells, antibodies, and MBCs were significantly increased after 3–4 repeated vaccinations and maintained at high levels throughout seasons 2012 and 2013. The cross-reactive IFN-γ-secreting CD4^+^ cells recognizing conserved viral external or internal epitopes were also maintained throughout 2012 and 2013. Repeated vaccination improved the multifunctional memory CD4^+^ responses. Particularly, the IFN-γ^+^TNF-α^+^CD4^+^ T cells were boosted following each vaccination. HI antibodies were significantly induced after each vaccination over 5 years. Our findings indicate a broad impact of repeated annual vaccination, even with the same vaccine component, on the influenza-specific T-cell and humoral immunity and support the continuing recommendation of annual influenza vaccination.

## Influenza vaccination: Repeat vaccination augments human immune responses

Despite the widespread implementation of annual influenza vaccination, the effect of repeated vaccinations on the immune response in subsequent seasons is poorly understood. A team lead by Rebecca Jane Cox at the University of Bergen examine the humoral and T cell response elicited by 2009’s H1N1pdm09 seasonal vaccine. Since the H1N1pdm09 strain continued to circulate in subsequent years it was included in later vaccine formulations and the authors could therefore examine the effects of repeated annual vaccination over multiple seasons. They observed that H1N1pdm09-specific polyfunctional T cell responses and antibodies were enhanced by multiple annual vaccinations. In particular, T cells showed progressive skewing to IFN-γ^+^TNF^+^ double producers but the magnitude of the T cell response tended to plateau after 3–4 repeated vaccinations. The findings suggest that including the same component in subsequent annual vaccines can significantly impact the influenza immune response.

## Introduction

Influenza virus remains a major health challenge due to its continuous ability to evade the hosts’ immunity. Annual seasonal influenza vaccination is the main method of prophylaxis for high-risk populations and healthcare workers (HCWs) providing protection against influenza A/H1N1, A/H3N2, and B viruses.^1^ In 2009, a novel H1N1 virus (H1N1pdm09) emerged and caused the first pandemic of the twenty-first century. HCWs were prioritized for pandemic vaccination to protect their patients and themselves, and maintain the integrity of the healthcare system.^2^ The AS03-adjuvanted monovalent H1N1pdm09 vaccine was used during the pandemic in Norway and provided protection against laboratory-confirmed influenza infection and hospitalization.^3^ The H1N1pdm09 virus continued to circulate after 2009 replacing earlier H1N1 strains and was therefore included in the seasonal vaccines as the A/H1N1 component during seasons 2010−2016.

Antibodies directed against the main viral surface glycoprotein, hemagglutinin (HA), can neutralize the influenza virus. The hemagglutination inhibition (HI) assay has been widely used to evaluate the HA-specific antibody responses. An HI titer of 40 is established as a surrogate correlate of protection against influenza at a 50% protective threshold.^4^ Inactivated influenza vaccines are standardized by the quantity of HA of each strain and induce HI antibodies after vaccination. Moreover, T cells have recently gained more recognition for their protective roles. Preexisting influenza-specific interferon (IFN)-γ-secreting CD4^+^ or CD8^+^ T cells can recognize conserved viral epitopes and provide cross-protection from heterosubtypic influenza A viruses, even in the absence of protective antibodies.^5–8^

Importantly, influenza vaccines have been used for decades; however, the long-term impact of repeated annual vaccination on antibody responses is not fully understood^9–11^ and there are limitations of our knowledge of its impact on T-cell responses. The emergence of the H1N1pdm09 virus and its inclusion as the A/H1N1 component in the seasonal vaccines for multiple years provided a unique opportunity to investigate the impact of repeated vaccination. Previously, we investigated the impact of repeated annual vaccination upon preexisting influenza-specific CD8^+^ and CD4^+^ T cells prior to two consecutive influenza seasons in HCWs who were either repeatedly vaccinated or only received a pandemic vaccination.^12^ In the current study, we further explored the impact of annual vaccination on T cells, particularly CD4^+^ T cells, and humoral immunity by assessing paired pre- and postvaccination T cell, antibody, and memory B-cell (MBC) responses in repeatedly vaccinated HCWs over 5 years. We have extended our previous findings to show that repeated annual vaccination with the same strain augmented both humoral and CD4 T-cell responses, maintained the cross-reactive IFN-γ-secreting CD4^+^ T cells recognizing viral external and internal epitopes, while increasing multifunctional memory CD4^+^ responses. Our findings have implications for the seasonal influenza vaccination strategy and vaccine development.

## Results

### Study population

**Fig. 1:**
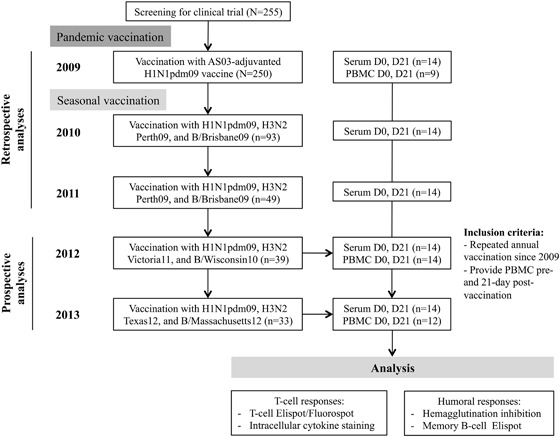
The study flow chart. Healthcare workers (HCWs) were vaccinated with a single dose of the AS03-adjuvanted monovalent pandemic H1N1pdm09 vaccine (European Clinical Trials Database, EudraCT 2009-016456-43; www.clinicaltrials.gov, NCT01003288). During 2010–2013 seasons, HCWs were vaccinated every year with the trivalent seasonal inactivated vaccines, containing the H1N1pdm09 virus as the A/H1N1 component during the whole study period. The A/H3N2 and B strains included in the seasonal vaccines changed between seasons during 2010–2013. The figure shows the inclusion criteria for this study, which were repeated annual vaccination between 2010 and 2013, and provision of peripheral blood monocular cells (PBMC) prevaccination (D0) and 21 days postvaccination (D21) in 2012 and 2013 seasons

Fourteen HCWs (mean age 41.2 years old, range 30–63 years), who received the AS03-adjuvanted pandemic vaccine in 2009 and subsequently annual seasonal vaccination during seasons 2010–2013, were included in this study (Fig. 1). Most HCWs (12/14) were female, worked on a clinical ward, and had history of previous seasonal vaccination before 2009 (Supplementary-Table 1).

### Maintenance of H1N1pdm09-specific IFN-γ-secreting T cells

The magnitude of preexisting and vaccine-induced H1N1pdm09-specific T cells was assessed before and after seasonal vaccination in 2012 and 2013 by measuring the IFN-γ-, interleukin(IL)-2-, or double-cytokine IFN-γIL-2 secretion against the split H1N1pdm09 virus and the H1N1-specific internal protein peptide pools: matrix 1 (M1), nucleoprotein (NP), and polymerase-basic 1 (PB1).

**Fig. 2:**
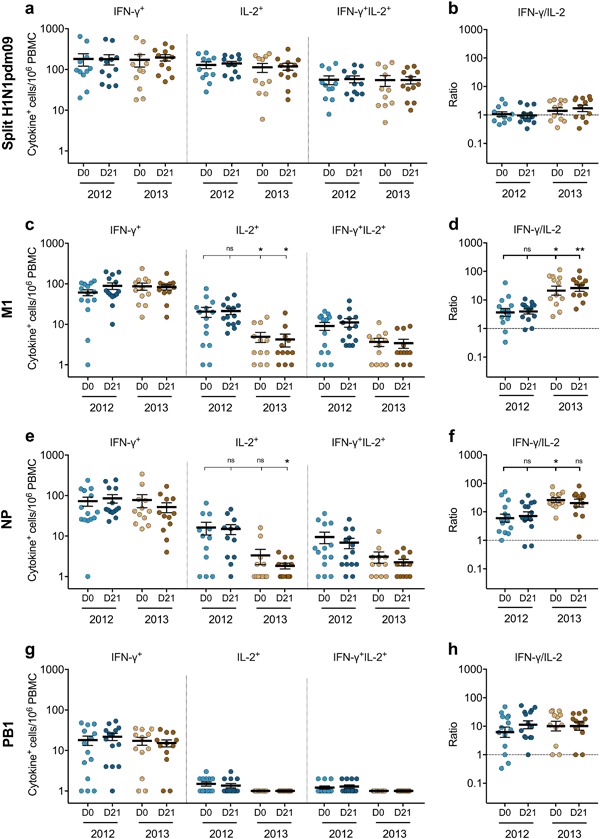
The magnitude of H1N1pdm09-specific T-cell responses during seasons 2012 and 2013. The single IFN-γ- or IL-2-, or double-cytokine IFN-γIL-2-secreting T cells against **a** the split H1N1pdm09 virus, the H1N1-specific viral internal proteins: **c** M1, **e** NP, or **g** PB1 prevaccination (D0) (light) and at 21 days postvaccination (D21) (dark) were measured in the T-cell FluoroSpot assay. Each symbol represents one individual’s response with the horizontal lines representing the mean magnitudes of cytokine-secreting cells per 10^6^ PBMC with standard error of the mean (s.e.m.). The IFN-γ to IL-2 ratio was calculated for each participant at each time point and the mean ratios with s.e.m. are shown against **b** the split H1N1pdm09 virus, the viral internal proteins: **d** M1, **f** NP, or **h** PB1. The dotted line indicates the IFN-γ/IL-2 ratio of 1 showing a balanced IFN-γ and IL-2 response. An IFN-γ/IL-2 ratio above or below 1 shows a predominant IFN-γ or IL-2 response, respectively. ^ns^*p* > 0.05, *p < 0.05, **p < 0.01

All cytokine-secreting T cells against the split virus were maintained at high levels throughout the two seasons, although no significant boost of these responses was observed after vaccination (Fig. 2a). The IFN-γ-secreting T cells recognizing the three internal proteins were maintained throughout 2012 and 2013 (Fig. 2c, e, g). However, we observed a decline in IL-2-secreting T cells recognizing M1 and NP after 1 year and a low but stable level of double-cytokine-secreting cells, leading to an increased IFN-γ/IL-2 ratio over time (Fig. 2d, f). Whereas IFN-γ dominated the responses against PB1, as there was almost no IL-2 or double-cytokine secretion (Fig. 2g, h). The dominant IFN-γ trend was not observed in the H1N1pdm09-specific T cells against the split virus (containing mainly surface glycoproteins), which had a balanced IFN-γ/IL-2 response (Fig. 2b).

### Maintenance of cross-reactive IFN-γ-secreting CD4^+^ T cells

**Fig. 3:**
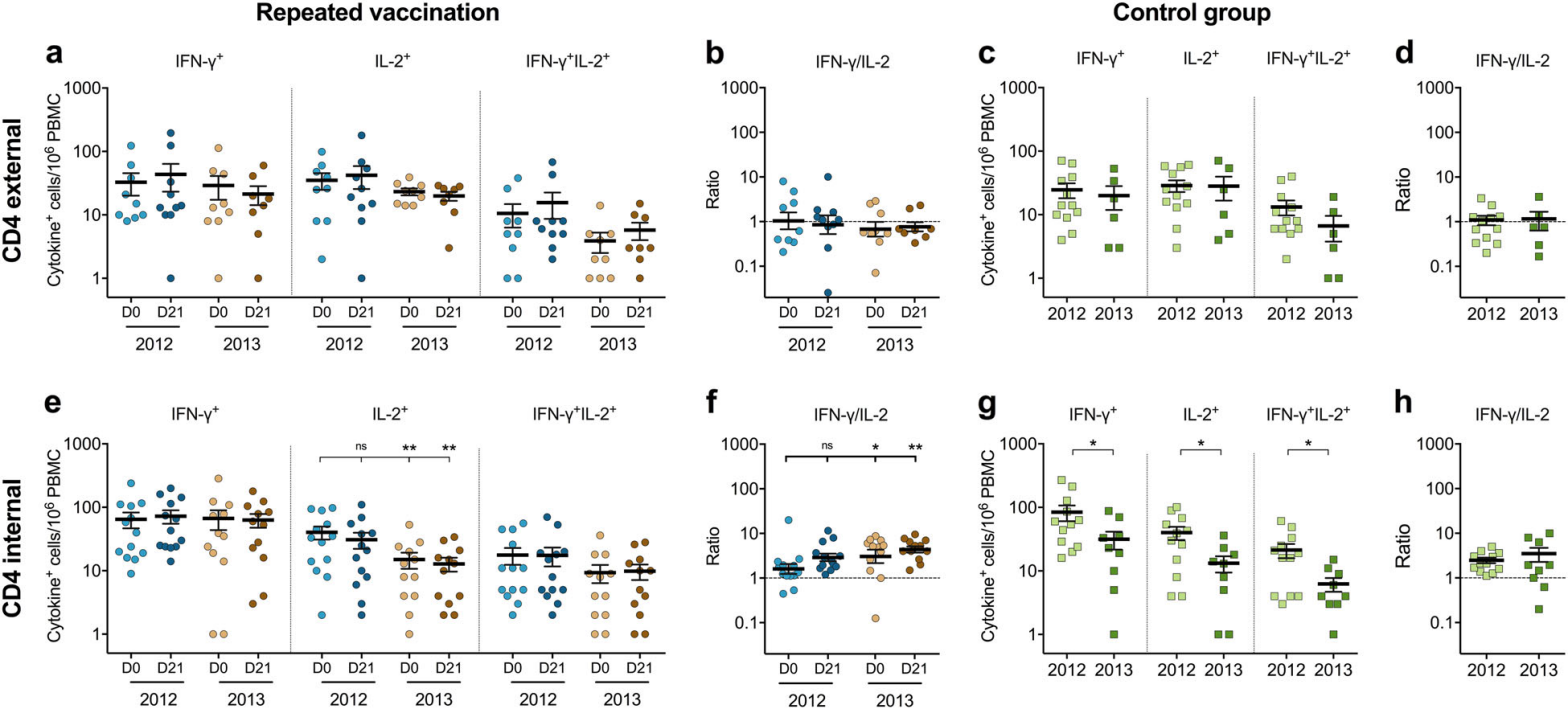
The magnitude of cross-reactive CD4^+^ T-cell responses during seasons 2012 and 2013. The single IFN-γ- or IL-2-, or double-cytokine IFN-γIL-2-secreting T cells against the CD4-specific conserved epitopes from viral external/internal proteins were measured in the T-cell FluoroSpot assay **a** and **e** prevaccination (D0) (light) and at 21 days postvaccination (D21) (dark) in HCWs with annual repeated vaccination (round) or **c** and **g** prior to the influenza season in 2012 (light) and 2013 (dark) in a control group of HCWs who were only vaccinated with the AS03-adjuvanted pandemic H1N1pdm09 vaccine in 2009 (square).^12^ Each symbol represents one individual’s response with the horizontal lines representing the mean magnitudes of cytokine-secreting cells per 10^6^ PBMC with standard error of the mean (s.e.m.). The IFN-γ to IL-2 ratio was calculated for each participant at each time point and the mean ratios with s.e.m. are shown against the CD4-specific external/internal epitopes **b** and **f** in HCWs with repeated vaccination or **d** and **h** a control group. The dotted line indicates the IFN-γ to IL-2 ratio of 1 showing a balanced IFN-γ and IL-2 response. An IFN-γ to IL-2 ratio above or below 1 shows a predominant IFN-γ or IL-2 response, respectively. ^ns^*p* > 0.05, *p < 0.05, **p < 0.01

The influenza-specific cross-reactive CD4^+^ T-cell responses were investigated using separate CD4 external or internal peptide pools. We found that the cross-reactive IFN-γ- or IL-2-secreting CD4^+^ T cells against external epitopes were maintained, while there was a trend of increased double-cytokine responses after vaccination (*p* = 0.094) (Fig. 3a). The cross-reactive IFN-γ- or double-cytokine-secreting CD4^+^ T cells against internal epitopes were maintained throughout the two seasons, whereas the IL-2-secreting cells declined after vaccination in 2012 (*p* = 0.088) and further in 2013 (*p* = 0.003) (Fig. 3e), resulting in the shift toward IFN-γ dominance over time (Fig. 3f). However, the trend of predominant IFN-γ was not observed in the cross-reactive responses to external epitopes (Fig. 3b).

To further examine this trend, T-cell responses were assessed in a control group of HCWs who were only vaccinated with the AS03-adjuvanted H1N1pdm09 vaccine in 2009 and received no further seasonal vaccinations. We have previously reported a decline in all cytokine-secreting cross-reactive CD4^+^ T cells against internal epitopes and H1N1pdm09-specific T cells after a year in these HCWs (Fig. 3g and Supplementary-Fig. 1a).^12^ We assessed the CD4^+^ T cells against external epitopes and observed a decline in double-cytokine secretion after a year, although not significant (Fig. 3c). A balanced IFN-γ/IL-2 response against the CD4 external epitopes and split H1N1pdm09 virus was found in this control group (Fig. 3d and Supplementary-Fig. 1b). However, no trend of increasing IFN-γ/IL-2 ratio in the CD4^+^ T cells specific for viral internal epitopes was observed, supporting the impact of repeated vaccination in favoring IFN-γ responses (Fig. 3h).

### Vaccine-induced boost of multifunctional IFN-γ^+^ and memory CD4^+^ T cells

**Fig. 4:**
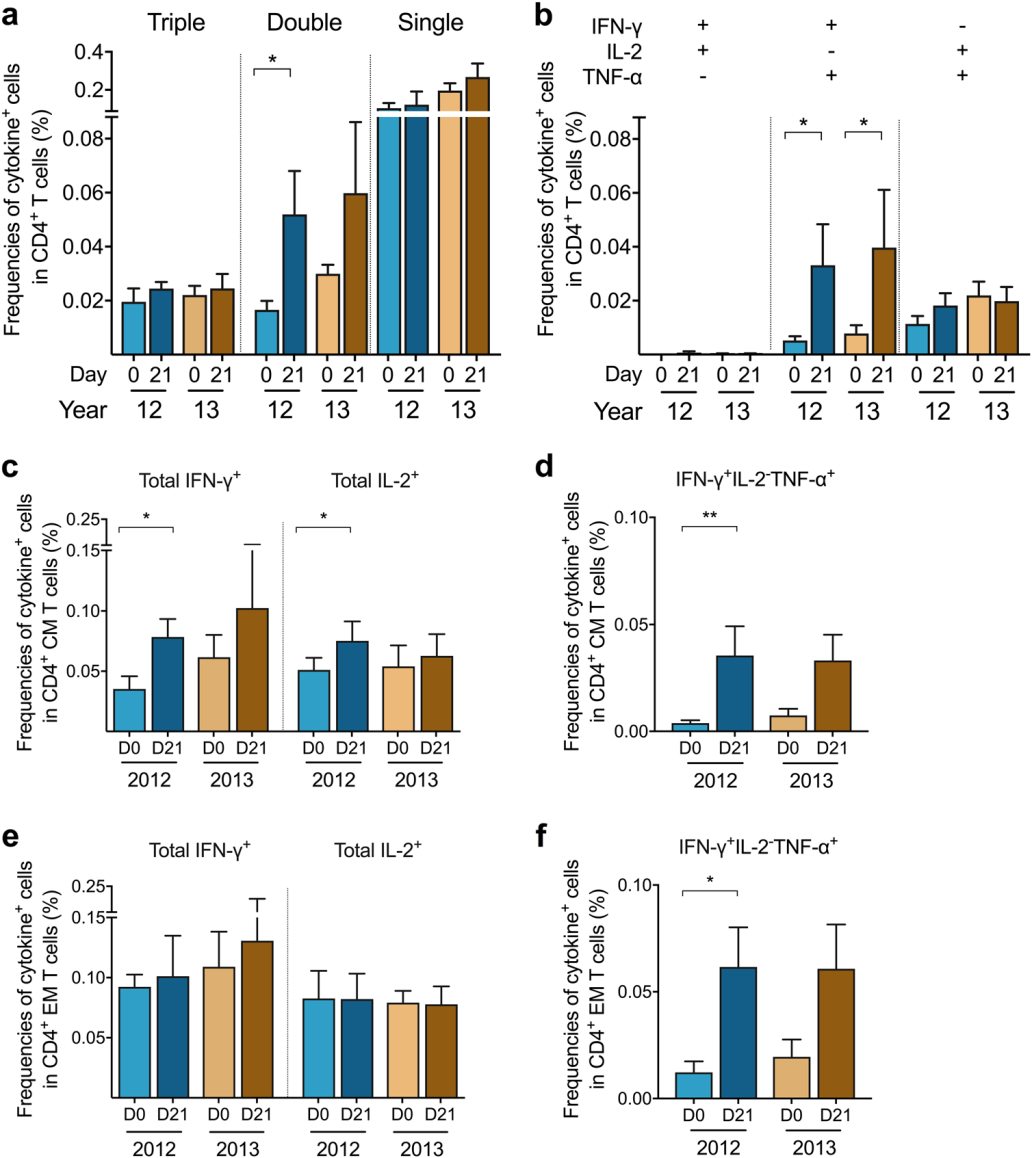
The quality of CD4^+^ T-cell responses during seasons 2012 and 2013. The cytokine profile of H1N1pdm09-specific CD4^+^ T cells was assessed by IFN-γ/IL-2/TNF-α intracellular cytokine-staining (ICS) assay prevaccination (D0) (light) and at 21 days postvaccination (D21) (dark) in the 2012 and 2013 seasons. **a** The mean frequencies (%) of the triple producer (IFN-γ^+^IL-2^+^TNF-α^+^), double producers (IFN-γ^+^IL-2^+^, IFN-γ^+^TNF-α^+^, IL-2^+^TNF-α^+^), and single producers (IFN-γ^+^, IL-2^+^, TNF-α^+^) with standard error of the mean (s.e.m.) are shown as bars. **b** The mean frequencies with s.e.m. are presented for each of the three double-producer subsets. The memory CD4^+^ T-cell responses were assessed by differentiating the three memory subsets CD45RA^−^CCR7^+^ central memory (CM), CD45RA^−^CCR7^−^effector memory (EM), and CD45RA^+^CCR7^−^ late effector memory (EMRA), from CD45RA^+^CCR7^+^ naïve T cells within the CD4^+^ population, then IFN-γ^+^ or IL-2^+^ cells were gated within CD4^+^ CM or EM population (see gating strategy in Supplementary-Fig. 4). The mean frequencies with s.e.m. of the total IFN-γ- or IL-2-secreting CD4^+^ **c** CM or **e** EM T cells pre- and postvaccination in seasons 2012 and 2013 are shown. The mean frequencies with s.e.m. are presented for the IFN-γ^+^IL-2^−^TNF-α^+^ subset of the CD4^+^ **d** CM or **f** EM T cells. *p < 0.05, **p < 0.01

Next, we explored the quality of H1N1pdm09-specific CD4^+^ T cells before and after vaccination in 2012 and 2013 by assessing the cytokine profile and memory subsets against the split H1Npdm09 antigen in the intracellular cytokine-staining assay (*n* = 5). The cytokine profile included seven cytokine-secreting subsets: triple producer (IFN-γ^+^IL-2^+^ tumor-necrosis-factor (TNF)-α^+^), double producers (IFN-γ^+^IL-2^+^, IFN-γ^+^TNF-α^+^, IL-2^+^TNF-α^+^), and single producers (IFN-γ^+^, IL-2^+^, TNF-α^+^). Higher frequencies of double producers were found after vaccination in 2012 (*p* = 0.031) and 2013 (*p* = 0.173) (Fig. 4a). Detailed analysis of double producers showed that the frequencies of IFN-γ^+^TNF-α^+^CD4^+^ T cells were significantly boosted after each seasonal vaccination in 2012 (*p* = 0.021) and 2013 (*p* = 0.046) (Fig. 4b). The frequencies of prevaccination IL-2^+^TNF-α^+^CD4^+^ cells were higher in 2013 than 2012, although not significant (*p* = 0.08).

We assessed the CD45RA^−^CCR7^+^central memory (CM) and CD45RA^−^CCR7^−^effector memory (EM) CD4^+^ T-cell subsets secreting IFN-γ or IL-2. We found that the long-lived CD4^+^ CM T cells secreting IFN-γ (*p* = 0.042) or IL-2 (*p* = 0.038) were boosted after vaccination in 2012, waned throughout the year although persisting at comparatively higher levels than prevaccination (Fig. 4c). Following 2013 vaccination, these responses were boosted, but not significantly. The IFN-γ^+^ or IL-2^+^CD4^+^ EM T cells remained stable throughout the two consecutive seasons (Fig. 4e). Interestingly, higher frequencies of double producers IFN-γ^+^TNF-α^+^CD4^+^ CM (*p* = 0.009) and EM (*p* = 0.030) T cells were observed after vaccination in 2012, albeit not significant in 2013 (*p* = 0.223 and 0.079, respectively) (Fig. 4d, f).

### Enhanced quantity and quality of T cells after repeated vaccinations

**Fig. 5:**
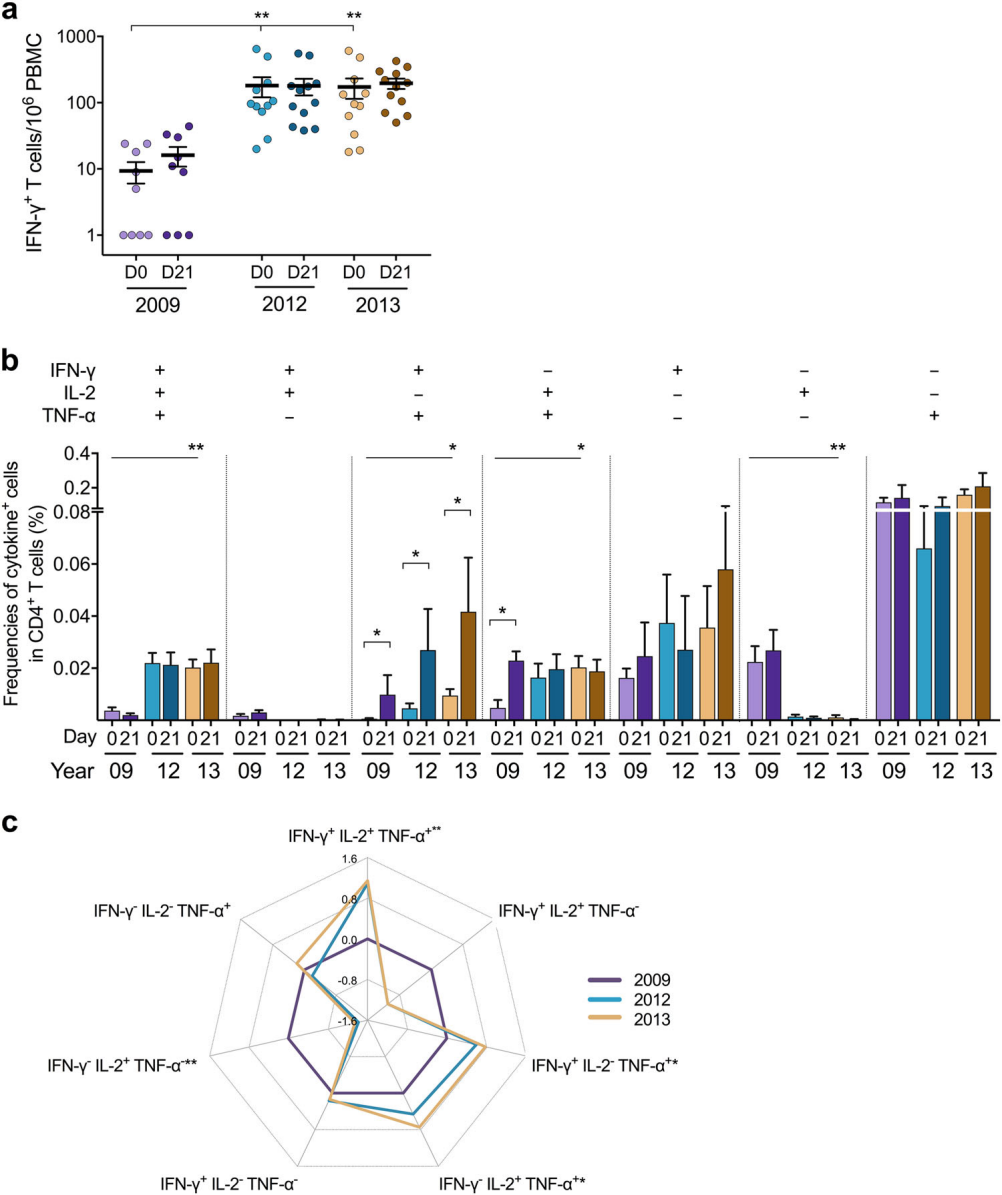
The impact of repeated annual vaccinations on the magnitude and the quality of H1N1pdm09-specific T cells over 5 years. **a** The magnitude of H1N1pdm09-specific IFN-γ-secreting T cells prevaccination (D0) (light) and at 21 days postvaccination (D21) (dark) were enumerated in the IFN-γ T-cell ELISpot assay in 2009 (*n* = 9), and the IFN-γ/IL-2 FluoroSpot assay in 2012 and 2013. Each symbol represents one individual’s response with the horizontal lines representing the mean magnitudes of IFN-γ-secreting cells per 10^6^ PBMC with standard error (s.e.m.). **b** The cytokine profile of H1N1pdm09-specific CD4^+^ T cells was assessed by the IFN-γ/IL-2/TNF-α intracellular cytokine-staining (ICS) assay pre- and postvaccination in 2009, 2012, and 2013. The mean frequencies (%) with s.e.m. of each of the seven cytokine-combination subsets are shown as bars. **c** Changes in the prevaccination cytokine profile of CD4^+^ T cells after 3–4 years of repeated vaccination. The fold-changes of responses between 2009 (reference) and 2012 or 2013 were calculated for each participant and the log-transformed means of fold-changes are presented in a radar chart. Value above or below 0 represents an increase or decline of response, respectively. *p < 0.05, **p < 0.01

We further investigated the long-term impact of repeated vaccination on T cells during the 5 study-years by retrospectively analyzing the H1N1pdm09-specific T-cell responses after pandemic vaccination in 2009^13^ and comparing to the response after seasonal vaccination in 2012 and 2013. Pre- and post-2009 pandemic vaccination data from nine HCWs who were subsequently annually vaccinated during 2010–2013 were included, of which four HCWs had repeated paired pre- and postvaccination data. The magnitude of prevaccination H1N1pdm09-specific IFN-γ-secreting T cells was significantly higher in 2012 (*p* = 0.016) and 2013 (*p* = 0.025) compared to pre-pandemic vaccination in 2009, although no significant boost was observed at 21 days postvaccination in these seasons (Fig. 5a, Supplementary-Fig. 2a).

By comparing cytokine profiles across these three seasons, we found a significant increase in multifunctional CD4^+^ T cells (secreting 2−3 cytokines) after repeated vaccinations (Fig. 5b, Supplementary-Fig. 2b), indicating an enhanced T-cell quality over the 5 years.^14,15^ The frequencies of triple producer (*p* = 0.008), and double producers IFN-γ^+^TNF-α^+^ (*p* = 0.018) and IL-2^+^TNF-α^+^ (*p* = 0.034) CD4^+^ T cells prevaccination were significantly higher in 2013 than in 2009. Interestingly, the IFN-γ^+^TNF-α^+^ and IL-2^+^TNF-α^+^CD4^+^ responses were boosted following 2009 vaccination (*p* = 0.031). In contrast, the frequencies of single IL-2-secreting T cells in the last two seasons were significantly lower than in 2009 (*p* = 0.008). The prevaccination cytokine-profile radar chart (Fig. 5c) shows that repeated vaccinations skewed the CD4^+^ T-cell responses toward IFN-γ dominance and the IL-2 responses were shifted from single to multifunctional IL-2 (double IL-2^+^TNF-α^+^ or triple IFN-γ^+^IL-2^+^TNF-α^+^ co-producers).

### Maintenance of high humoral responses after repeated vaccinations

**Fig. 6:**
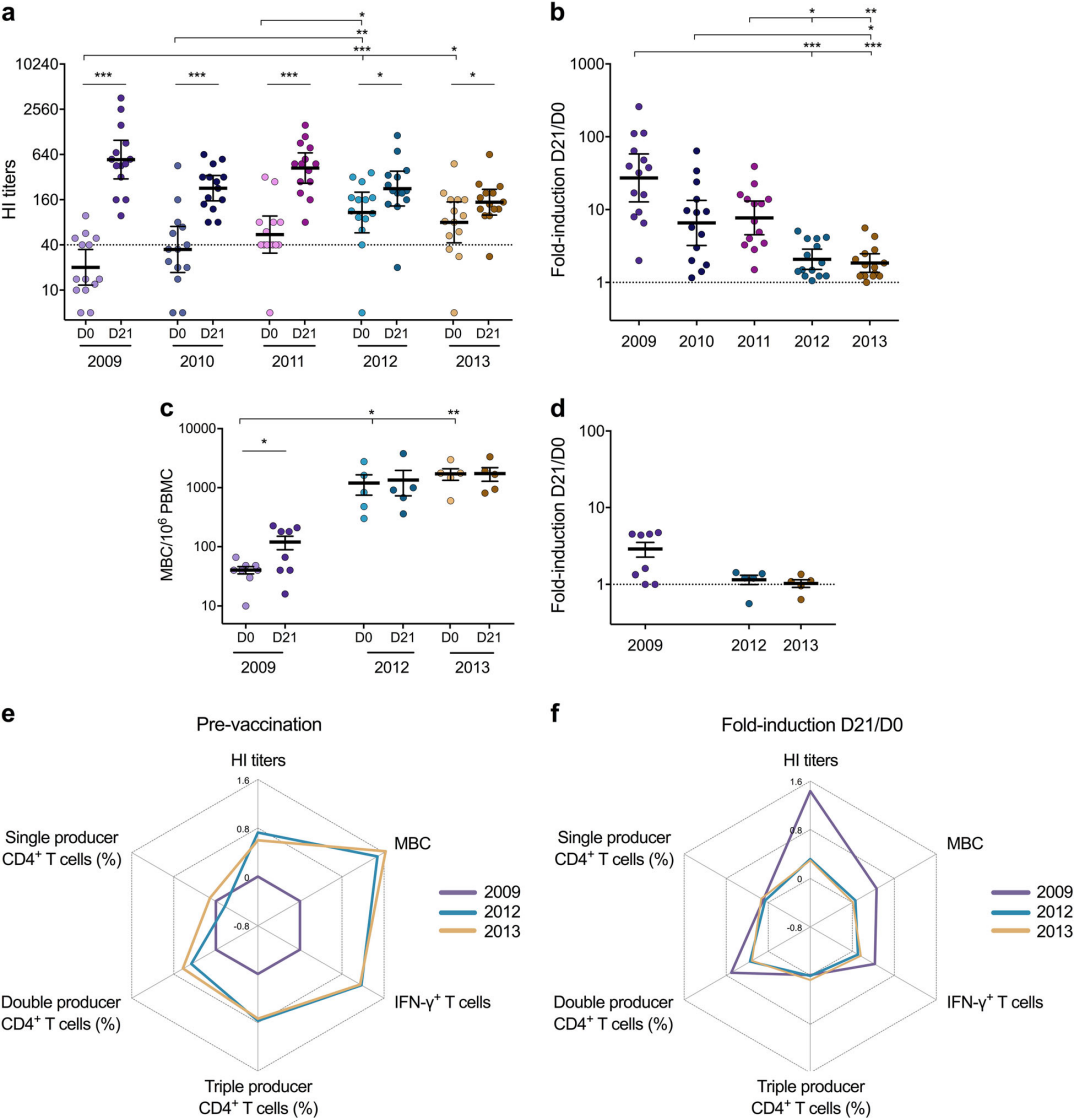
The overall impact of repeated annual vaccinations on T-cell and humoral responses. **a** The 5-year H1N1pdm09-specific antibody responses following pandemic and repeated seasonal vaccinations were measured in the hemagglutination inhibition (HI) assay. Each symbol represents one individual’s response with the horizontal lines representing the geometric mean HI titer with 95% confidence interval (CI) prevaccination (D0) (light) and at 21 days postvaccination (D21) (dark) each year. The dotted line indicates the 50% protective threshold defined as HI titer ≥ 40. **b** The HI antibody fold-induction between pre- and postvaccination (D21/D0) titers was calculated for each participant at each time point. The horizontal lines represent the geometric mean HI antibody fold-induction with 95% CI. **c** The H1N1pdm09-specific memory B-cell (MBC) responses pre- and postvaccination in seasons 2009, 2012, and 2013 were measured in the MBC ELISpot assay. The horizontal lines represent the mean magnitudes of the H1N1pdm09-specific MBCs per 10^6^ PBMC with standard error of the mean (s.e.m.). **d** The MBC fold-induction between pre- and postvaccination (D21/D0) was calculated for each participant at each time point. The horizontal lines represent the mean MBC fold-induction with s.e.m. **e** The fold-changes of prevaccination antibodies, MBCs, and T cells between 2009^13^ and 2012 or 2013 were calculated for each participant and the log-transformed means of fold-changes are presented in a radar chart. Value above or below 0 represents an increase or decline of response, respectively. **f** The log-transformed mean fold-induction of T-cell and humoral responses between pre- and postvaccination (D21/D0) each year (2009, 2012, and 2013) is presented in a radar chart. The higher the value is above 0, the higher the vaccine-induced response is after vaccination. *p < 0.05, **p < 0.01, ***p < 0.001

Throughout the 5 years of our study, the H1N1pdm09-specific HI antibodies were significantly boosted after each vaccination (Fig. 6a). After the second vaccination, HI antibodies persisted above the 50% protective threshold (HI titers ≥ 40) for 1-year postvaccination in all HCWs, except one who had chronic respiratory and neurological conditions. The prevaccination HI titers gradually increased from 2009 to 2012, then were maintained between 2012 and 2013. The antibody fold-induction between pre- and postvaccination titers was highest after the adjuvanted pandemic vaccination in 2009 then declined after multiple vaccinations (Fig. 6b). The fold-induction after seasonal vaccination in 2012 and 2013 was significantly lower than in earlier seasons.

We further assessed the H1N1pdm09-specific MBC responses in HCWs pre- and postvaccination in 2012 and 2013 (*n* = 5 due to limited PBMC availability), while the 2009 data were retrospectively analyzed from nine HCWs.^13^ The prevaccination H1N1pdm09-specific MBCs was significantly higher in 2012 and 2013 than in 2009 (Fig. 6c). Notably, MBCs were significantly boosted following pandemic vaccination in 2009 (*p* = 0.031), but not after seasonal vaccination in 2012 and 2013. The fold-induction after vaccination was higher in 2009 than in 2013 (*p* = 0.061) (Fig. 6d).

We investigated the overall impact of repeated vaccination against H1N1pdm09 on immune responses using radar charts. An increase in both humoral and T-cell immunity specific for H1N1pdm09 prevaccination were observed after 3−4 vaccinations (Fig. 6e). Interestingly, MBCs and double producers CD4^+^ T cells continue to increase from 2012 to 2013. The fold-induction of the vaccine-induced responses postvaccination was highest after adjuvanted pandemic vaccination in 2009 (Fig. 6f).

## Discussion

There is limited knowledge about the long-term impact of repeated annual influenza vaccination on the influenza-specific T-cell immunity despite decades of use of influenza vaccine. Here, we provide unique data on humoral and T-cell responses after repeated annual vaccination with the same H1N1pdm09 strain from its introduction in 2009 and over four subsequent seasons.

Repeated vaccinations resulted in an increase in both the quantity and the quality of H1N1pdm09-specific T-cell responses. One study suggested that >20 IFN-γ-secreting T cells/10^6^ PBMC as detectable protective influenza-specific T cells at a population level.^8^ In our repeatedly vaccinated HCW cohort, the prevaccination IFN-γ-secreting T cells increased significantly from non-detectable (5 cells/10^6^ PBMC) to persistently high numbers (95 cells/10^6^ PBMC) after 3−4 vaccinations, although we do not have T-cell data from 2010 and 2011 to confirm whether these cells increased continuously after each year of repeated vaccination until 2012 and 2013, similarly to the antibody responses. Whereas in the absence of repeated vaccination, a decline in H1N1pdm09-specific cytokine-secreting T cells from 2012 to 2013 was observed in single vaccinated individuals (Supplementary-Fig. 1a).^12^ Moreover, the quality of CD4^+^ T cells was improved with higher multi-cytokine-secreting responses, associated with superior function compared to single-cytokine producers.^14,15^ The boost of IFN-γ^+^TNF-α^+^CD4^+^ responses after each vaccination in 2009, 2012, and 2013 suggests a continuous increase of these responses. We hypothesize that the magnitude of T cells may reach a plateau after 3−4 repeated vaccinations, like the antibodies, while the quality of T cells continues to improve. Although this study did not assess the quality of antibodies, our earlier report showed that repeated vaccination maintained the antibody avidity.^16^ This implies that repeated vaccination may have a broader impact on immune responses, which are not usually assessed in conventional vaccine immunogenicity studies.^17^ Future studies should therefore include diverse immunological assessments to better understand vaccine immunogenicity in populations with different influenza exposure backgrounds.

We demonstrated that the H1N1pdm09-specific HI antibodies were boosted after each vaccination during the 5 study-years. The prevaccination antibodies increased each year until 2012 and 2013 persisting above the 50% protective threshold, whereas the antibody fold-induction postvaccination declined and was lowest in the last two seasons compared to previous seasons. This suggests that repeated vaccinations sustain high protective antibody levels rather than boosting them. The long-term antibody persistence is probably due to activation of MBCs that can undergo continuous proliferation and differentiation to maintain constant levels of plasma cells and antibodies.^18^ T cells also contribute to induction and maintenance of antibodies by providing help to activate naïve and MBCs.^19^ This hypothesis was supported by the significantly higher H1N1pdm09-specific MBCs and T cells observed after 3−4 repeated vaccinations. Moreover, the decline in antibody fold-induction observed in later seasons may be due to the presence of high prevaccination HI antibodies, which may block the vaccine epitopes, resulting in a reduction of B- and T-cell activation,^20–22^ and therefore limit the boosting ability of subsequent vaccinations.

The controversy of annual influenza vaccination policy involves conflicting reports on the impact of repeated vaccination, which reported either no significant interference or a reduction in antibody responses and vaccine effectiveness (VE).^9–11,23,24^ In studies that found that repeated vaccination led to decreased protection, this was mainly related to the H3N2 viruses. The H3N2 subtype has been associated with lower VE and undergoes more frequent antigenic changes compared to the H1N1 and B viruses included in the seasonal vaccines.^25^ A recent study investigating the impact of repeated vaccination against H1N1pdm09 on VE suggests that VE was highest after 2−3 vaccinations and reduced in vaccinees immunized with >3 vaccinations.^11^ Our study did not find a reduction in H1N1-specific immune responses after 3−4 vaccinations, although protection was not assessed. Our findings show the maintenance of H1N1pdm09-specific antibodies, MBCs and T cells at high levels with improved quality of CD4^+^ T cells after 3−4 repeated vaccinations. However, enhanced T-cell responses do not prevent infection, the outcome in most studies investigating protection use, but reduce the severity of illness.^5–8^ We suggest that broader outcome measures of protection, such as severity scores or time to recover, should be incorporated into future studies together with various immunological assessments to better evaluate the impact of repeated vaccination. Taken together, we support the continuation of the current recommendation of annual influenza vaccination, even with the same vaccine component, to provide protection against all circulating seasonal strains. Our findings point to the importance of inclusion of influenza vaccination history, when evaluating vaccine immunogenicity and effectiveness.

Remarkably, the long-lived H1N1pdm09-specific CD4^+^ CM T cells were boosted after vaccination. Since CM cells can rapidly proliferate and differentiate into effector cells upon antigen encounter,^26^ the persistent CD4^+^ CM and EM responses following repeated vaccination may provide long-lasting protection. These findings agree with our previous report^12^ and provide an explanation for the long-term maintenance of IFN-γ^+^ and multifunctional CD4^+^ T cells in repeatedly vaccinated HCWs, which was not observed in HCWs immunized with only pandemic vaccination. Our findings suggest that repeated vaccination optimizes the quantitative responses while shifting the influenza-specific immunity towards long-term memory and multifunctional responses. However, this hypothesis needs to be verified with the other influenza vaccine strains, H3N2 and B viruses, which change more frequently than H1N1 viruses.

Influenza-specific CD4^+^ T cells can recognize conserved epitopes from heterosubtypic influenza A viruses and provide cross-protection^5^ and are boosted after a single vaccination.^13,27,28^ We extended these findings by separately investigating the cross-reactive CD4^+^ responses to conserved viral external or internal epitopes following repeated vaccination, as these cells may be phenotypically different with distinct potential functions.^29–32^ After 3−4 repeated vaccinations, a decline in IL-2 responses resulting in a trend of increased IFN-γ dominance against viral internal epitopes was observed, which agrees with previous reports.^33,34^ Long-term exposure to influenza through vaccination or infection may shape the T-cell responses towards conserved epitopes that are repeatedly recognized by influenza-specific memory T cells. We hypothesize that these responses are multifunctional with predominantly IFN-γ and a low level of IL-2. Interestingly, analyses of the quality of H1N1pdm09-specific CD4^+^ T cells after repeated vaccinations provided a potential explanation supporting this hypothesis. We found the boosting of IFN-γ^+^TNF-α^+^CD4^+^ T-cell responses following each vaccination, the increased multi-cytokine-secreting and decreased single IL-2-secreting cells after 3−4 repeated vaccinations. The ratio of IFN-γ to IL-2-secreting cells, calculated using the cytokine profile data showed that the H1N1pdm09-specific CD4^+^ T cells remained IL-2 enriched in 2009 but changed toward predominantly IFN-γ secretion in 2012 and 2013 (Supplementary-Fig. 3). However, whether this IFN-γ dominance in cross-reactive CD4^+^ T cells indicates a greater potential for help and/or other functions will need to elucidate in further studies.

We faced difficulties due to the long follow-up period, such as losing participants and missing samples. The strict inclusion criteria that only HCWs repeatedly vaccinated for 5 years greatly limited the sample size of our study. However, this design allows us to study the long-term impact of repeated vaccinations on immune responses while clarifying the effect of vaccination sequence without the bias of missing vaccination history. As HCWs who were repeatedly vaccinated for 5 years were identified in the last two seasons, the T-cell responses in 2009 were retrospectively evaluated. Although the low number of participants with accessible data and the lack of 2010 and 2011 information on T cells limited our observation, the immune responses after repeated vaccinations were undoubtedly enhanced. As memory T-cell responses are rapidly generated, we cannot dismiss the possibility of T-cell expansion at 7–14 days,^13^ time points that were not investigated in this study. Further studies are required to confirm our findings in larger populations with documented vaccination history, and to evaluate both antibody and T-cell responses against other vaccine strains after repeated annual vaccination.

In conclusion, we provide a unique overview of the long-term impact of repeated annual influenza vaccination against the same vaccine strain on humoral and T-cell immunity. Repeated vaccinations with H1N1pdm09 not only maintained the high magnitude of strain-specific HI antibodies and T cells, and cross-reactive IFN-γ^+^CD4^+^ T cells, but also increased MBC and multifunctional CD4^+^ T-cell responses. This study highlights a broad immunological impact of repeated vaccination and supports the current recommendation of annual seasonal influenza vaccination. Our findings suggest that routine collection of influenza vaccination history and diverse immunological approaches should be included when evaluating vaccine immunogenicity and effectiveness.

## Methods

### Study population and sampling

An open-label 5-year extension of a single-arm clinical trial was conducted in HCWs (Haukeland University Hospital, Norway) vaccinated with the 2009 AS03-adjuvanted pandemic H1N1pdm09 vaccine (www.Clinicaltrials.gov, NCT01003288). The study was approved by the regional ethics committee (REKVest-2012/1772) and the Norwegian Medicines Agency.^35^ All participants provided written informed consent before inclusion and new consent for the follow-up blood samples. During 2010–2013, HCWs were annually vaccinated with the non-adjuvanted seasonal trivalent inactivated vaccine (Vaxigrip, Sanofi Pasteur or Influvac, Abbott Laboratories) containing the H1N1pdm09 as the A/H1N1 component and different A/H3N2 and B viruses (Fig. 1). Serum samples were collected pre- and 21 days postvaccination for each season from 2009 to 2013. HCWs who provided additional PBMC samples pre- and 21-day postvaccination in 2012 and 2013 were included in this study.

Sera were separated from clotted blood and stored at −80 °C until analyzed. PBMC were isolated and cryopreserved at −150 °C in 90% fetal bovine serum/10% dimethyl sulfoxide until analyzed.^12^

### Hemagglutination inhibition (HI) assay

Receptor destroying enzyme-treated sera were tested in duplicate with 0.7% turkey red blood cells (TRBC) and eight HA units of inactivated A/California/07/2009 (H1N1) antigen, as described previously.^35^ The HI titer was the reciprocal of the highest serum dilution causing 50% inhibition of hemagglutination. Titers <10 were assigned a value of 5 for calculation purposes. Sera <40 were screened for nonspecific binding and preadsorbed with TRBC before re-analyzing.

### T-cell FluoroSpot assay

PBMC were stimulated with the split H1N1pdm09 antigen, the H1N1-specific M1, NP, or PB1 peptide pools (BEI Resources), or the conserved CD4 internal or external peptide pools to measure the IFN-γ- and/or IL-2-secreting T-cell responses, as described earlier.^12,36^ The M1, NP, or PB1 peptide pools included overlapping epitopes that covered the complete sequence of the three proteins. The CD4 peptide pools were chemically synthesized and consisted of HLA−class-II-restricted T-cell epitopes from internal or external viral proteins that are conserved among influenza A subtypes with high prevalence and HLA−supertype coverage (Supplementary-Tables 2, 3).^36^ Cytokine-secreting cells were counted and the background from non-stimulated cells was subtracted from stimulation responses.

### Intracellular cytokine-staining (ICS) assay

PBMC were stimulated with the split H1N1pdm09 antigen to measure the IFN-γ-, IL-2- and/or TNF-α-secreting CD4^+^ T-cell responses, as described previously.^12^ Data were acquired on an LSRFortessa flow cytometer and analyzed in FlowJo version-10 (see Supplementary-Fig. 4 for the gating strategy).

### Memory B-cell (MBC) ELISpot assay

The H1N1pdm09-specific IgG^+^MBC responses were measured by the ELISpot assay, as described elsewhere.^37^

### Statistical analyses

Comparisons of the T-cell responses assessed in the FluoroSpot or ICS assays in 2012 and 2013 and the 5-year log-transformed HI antibody titers were performed using the nonparametric repeated-measure Friedman test, followed by the Dunn−Bonferroni post-hoc test. The retrospective data for IFN-γ-secreting T cells, cytokine profile and MBCs in 2009 were compared to the prospective data in 2012 or 2013 using the nonparametric Kruskal−Wallis or Mann−Whitney test, as appropriate, with Bonferroni correction. Adjusted *p* values < 0.05 were considered statistically significant. Analyses were performed in SPSS-Statistics version-24 and visualized in Prism version-7.

### Data availability

The data that support the findings of this study are available from the corresponding author upon reasonable request.

## Electronic supplementary material

Supplementary figures and tables

Clinical trial protocol for 5 year follow up

## Electronic supplementary material

**Supplementary information** accompanies the paper on the *npj Vaccines* website (10.1038/s41541-018-0069-1).
