# Publisher Correction: Augmented CD4^+^ T-cell and humoral responses after repeated annual influenza vaccination with the same vaccine component A/H1N1pdm09 over 5 years

**DOI:** 10.1038/s41541-018-0081-5

**Published:** 2018-09-11

**Authors:** Mai-Chi Trieu, Fan Zhou, Sarah Larteley Lartey, Saranya Sridhar, Siri Mjaaland, Rebecca Jane Cox

**Affiliations:** 1 The Influenza Centre, Department of Clinical Science, University of Bergen, Bergen, Norway; 2 K.G. Jebsen Centre for Influenza Vaccine Research, Department of Clinical Science, University of Bergen, Bergen, Norway; 3 Jenner Institute, University of Oxford, Oxford, UK; 4 Department of Infectious Disease Immunology, Norwegian Institute of Public Health, Oslo, Norway; 5 Department of Research and Development, Haukeland University Hospital, Bergen, Norway; 6 Present Address: Sanofi Pasteur, 1541 Avenue Marcel Mérieux, 69280 Marcy l’Etoile, France

**Correction to**: *npj Vaccines* 10.1038/s41541-018-0069-1, Published online 14 August 2018

In the original published version of this Article, the text for the “Editorial Summary” was mistakenly incorporated at the end of the abstract. The HTML and PDF versions of the paper have been corrected.

