# Supplementary material for: Augmented CD4+ T-cell and humoral responses after repeated annual influenza vaccination with the same vaccine component A/H1N1pdm09 over 5 years
Source: NPJ Vaccines. 2018 Aug 14;3:37. doi: 10.1038/s41541-018-0069-1 (PMC6092382; doi:10.1038/s41541-018-0069-1)
Supplement: Supplementary file 1 — Supplementary figures and tables [file 41541_2018_69_MOESM1_ESM.docx]

**Augmented CD4^+^ T-cell and humoral responses after repeated annual influenza vaccination with the same vaccine component A/H1N1pdm09 over five years**

Mai-Chi Trieu^1,2,*^, Fan Zhou^1,2^, Sarah Larteley Lartey^1,2^, Saranya Sridhar^3^, Siri Mjaaland^2,4^, Rebecca Jane Cox^1,2,5,*^

**Supplementary files**

Supplementary-Figure 1………………………………….……………………………2

Supplementary-Figure 2………………………………………………………….……4

Supplementary-Figure 3……………………………………………………….………5

Supplementary-Figure 4…………………………………….…………………………6

Supplementary-Table 1………………………………………..……………………….8

Supplementary-Table 2……………………………..………..………………………...9

Supplementary-Table 3……………………………..………..……………………….10

**Supplementary-Figure 1:** The magnitude of H1N1pdm09-specific T-cell responses in 2012 and 2013. (a) The single IFN-γ- or IL-2-, or double-cytokine IFN-γIL-2-secreting T cells against the split H1N1pdm09 virus were measured in the T-cell FluoroSpot assay prior to the influenza season in 2012 (light) and 2013 (dark) in a control group of HCWs who were only vaccinated with the adjuvanted pandemic vaccine in 2009.^12^ Each symbol represents one individual’s response with the horizontal lines representing the mean magnitudes of cytokine-secreting cells per 10^6^ PBMC with standard error of the mean (s.e.m). (b) The IFN-γ to IL-2 ratio was calculated for each HCW at each time point against the split H1N1pdm09 virus and the mean ratios with s.e.m are presented. The dotted line indicates the IFN-γ/IL-2 ratio of 1 showing a balanced IFN-γ and IL-2 response. An IFN-γ/IL-2 ratio above or below 1 shows a predominant IFN-γ or IL-2 response, respectively. Comparisons of T-cell responses between 2012 and 2013 were performed using the non-parametric paired-sample Friedman test, followed by the Dunn-Bonferroni post-hoc test. *p<0.05.

**Supplementary-Figure 2**: The impact of repeated annual vaccinations on the magnitude and the quality of H1N1pdm09-specific T cells in the same healthcare workers over 5 years (n=4). (a) The magnitude of H1N1pdm09-specific IFN-γ-secreting T cells pre-vaccination (D0) (light) and at 21 days post-vaccination (D21) (dark) were enumerated in the IFN-γ T-cell ELISpot assay in 2009, and the IFN-γ/IL-2 FluoroSpot assay in 2012 and 2013. Each symbol represents one individual’s response with the horizontal lines representing the mean magnitudes of IFN-γ-secreting cells per 10^6^ PBMC with standard error of the mean (s.e.m). (b) The cytokine profile of H1N1pdm09-specific CD4^+^ T cells were assessed by IFN-γ/IL-2/TNF-α intracellular cytokine staining (ICS) assay pre- and post-vaccination in 2009, 2012 and 2013. The mean frequencies (%) with s.e.m of each of the 7 cytokine-combination subsets are shown as bars.

**Supplementary-Figure 3**: The IFN-γ to IL-2 ratio of cytokine-secreting CD4^+^ T-cell responses against H1N1pdm09 vaccine antigen in 2009, 2012 and 2013 assessed by the intracellular cytokine-staining (ICS) assay. The IFN-γ/IL-2 ratio was calculated by dividing the frequency of total IFN-γ^+^CD4^+^ T cells to the frequency of total IL-2^+^CD4^+^ T cells for each HCW at each time point. Horizontal lines represent the mean IFN-γ/IL-2 ratios with standard error of the mean (s.e.m) pre-vaccination (D0) (light) and at 21 days post-vaccination (D21) (dark) in 2009, 2012 and 2013. The dotted line indicates the IFN-γ/IL-2 ratio of 1 showing a balanced IFN-γ and IL-2 response. An IFN-γ/IL-2 ratio above or below 1 shows a predominant IFN-γ or IL-2 response, respectively. Comparisons of T-cell response ratio between 2009 and 2012 or 2013 were performed using the non-parametric Kruskal-Wallis test, followed by the Dunn-Bonferroni post-hoc test. No significant result was found.

**Supplementary-Figure 4:** The gating strategy for memory CD4^+^ T-cell subsets and cytokine responses used in the intracellular cytokine-staining assay. Firstly, dead cells were excluded from the analyzed population using live/dead staining. Single cells and lymphocytes were identified based on the cell size and granularity using forward scatter (FSC) and side scatter (SSC) parameters. For further analysis, only CD3^+^ T-cells were included, and CD56^+^ NK-cells and CD19^+^ B-cells were excluded. Next, CD4^+^ and CD8^+^ T-cells were separated. To identify the frequencies of cytokine^+^ CD4^+^ cells, total IFN-γ^+^, IL-2^+^ or TNF-α^+^ cells were gated from the CD4^+^ population. Boolean analysis was used to assess each cytokine combination response, triple producer (IFN-γ^+^IL-2^+^TNF-α^+^), double producers (IFN-γ^+^IL-2^+^, IFN-γ^+^TNF-α^+^, IL-2^+^TNF-α^+^) and single producers (IFN-γ^+^, IL-2^+^, or TNF-α^+^). To properly differentiate the four main memory subsets CD45RA^-^CCR7^+^ central memory (CM), CD45RA^-^CCR7^-^ effector memory (EM), CD45RA^+^CCR7^-^ late effector memory (EMRA), and CD45RA^+^CCR7^+^ naïve (NA) within the CD4^+^ cells, the gating for CD45RA and CCR7 markers were optimized using the fluorescence-minus-one (FMO) controls containing all the fluorochromes in the T-cell panel except for the measured one. Memory subset responses were assessed in 2 ways. (i) The memory subset compartment among cytokine^+^CD4^+^ cells – gated for CD45RA and CCR7 markers within IFN-γ^+^ or IL-2^+^CD4^+^ cells, and the four memory subsets as a proportion of 100%. (ii) The frequencies of cytokine^+^ cells among the memory CD4^+^ subsets – gated for CD45RA and CCR7 markers within CD4^+^ cells, then within each memory subset gated for IFN-γ^+^ or IL-2^+^ cells.

**Supplementary-Table 1:** The demographic and clinical characteristics of the fourteen healthcare workers who received the AS03-adjuvanted H1N1pdm09 vaccine in 2009 and repeated annual influenza vaccination in all subsequent seasons during 2010-2013.

| Characteristic (N=14) | Number | Percentage (%) |
| --- | --- | --- |
| Mean age (year range) | 41.2 (30-63) | - |
| Gender (female) | 12 | 86 |
| Work on a clinical ward | 12 | 86 |
| Previous seasonal vaccination | 12 | 86 |
| High-risk conditions^1^ | 2 | 14 |

^1^ One HCW with diabetes and one HCW with chronic respiratory and neurological diseases

**Supplementary-Table 2**: The influenza-specific CD4 external peptide pool used for stimulation of peripheral blood mononuclear cells in the FluoroSpot assay. The peptide pool contains an optimal combination of HLA class II-restricted T-cell epitopes from viral surface proteins conserved among influenza A subtypes.^37^ Peptide sequence were downloaded from the Immune Epitope Database (IEDB) and selected one by one by an algorithm ranking the score of prevalence, conservancy and HLA supertype coverage until reached the optimal number. Each peptide was chemically synthesized (Mimotopes, Australia) and pooled together at a concentration of 2μg/ml.

| **Order** | **Epitope ID** | **Peptide** | **Length** | **Protein** | **Preval-ence** | **Conser-vancy** | **S-type coverage** |
| --- | --- | --- | --- | --- | --- | --- | --- |
| 2 | 122152 | NAELLVLMENERTLDFHDSN | 20 | HA | 0.55 | 0.26 | 1.00 |
| 3 | 50489 | QDLEKYVEDTKIDLWSYNAELLVALENQHTIDLTDS | 36 | HA | 0.72 | 0.16 | 1.00 |
| 8 | 51554 | QLSSVSSFERFEIFPKESSW | 20 | HA | 0.29 | 0.13 | 0.71 |
| 9 | 73603 | YDVPDYASLRSLVASS | 16 | HA | 0.50 | 0.23 | 0.71 |
| 13 | 97325 | GDVFVIREPFISCSH | 15 | NA | 0.07 | 0.39 | 0.57 |
| 17 | 51780 | QNVNRITYGACPRYVKQNTLKLATGMRNVPEKQT | 34 | HA | 0.76 | 0.10 | 0.71 |
| 21 | 36498 | LIEKTNEKFHQIEKEFSEVEGRIQDLEKYVEDTKI | 35 | HA | 0.52 | 0.10 | 0.86 |
| 23 | 48237 | PKYVKQNTLKLAT | 13 | HA | 0.75 | 0.10 | 0.71 |
| 28 | 22098 | GRIQDLEKYVEDTKIDLWS | 19 | HA | 0.29 | 0.23 | 0.43 |
| 32 | 53588 | RENAEDMGNGCFKIYHKCDNACIGSIRNGTYDH | 33 | HA | 0.52 | 0.19 | 0.43 |

**Supplementary-Table 3**: The influenza-specific CD4 internal peptide pool used for stimulation of peripheral blood mononuclear cells in the FluoroSpot assay. The peptide pool contains an optimal combination of HLA class II-restricted T-cell epitopes from viral internal proteins conserved among influenza A subtypes.^37^ Peptide sequence were downloaded from the Immune Epitope Database (IEDB) and selected one by one by an algorithm ranking the score of prevalence, conservancy and HLA supertype coverage until reached the optimal number. Each peptide was chemically synthesized (Mimotopes, Australia) and pooled together at a concentration of 2μg/ml.

| **Order** | **Epitope ID** | **Peptide** | **Length** | **Protein** | **Preval-ence** | **Conser-vancy** | **S-type coverage** |
| --- | --- | --- | --- | --- | --- | --- | --- |
| 1 | 21087 | GLQRRRFVQNALNGNGDPNN | 20 | M1 | 0.42 | 0.90 | 0.71 |
| 4 | 41282 | MDVNPTLLFLKVPAQ | 15 | PB1 | 0.12 | 0.71 | 0.86 |
| 5 | 18366 | FWRGENGRKTRSAYERMCNILKGK | 24 | NP | 0.38 | 0.23 | 0.57 |
| 6 | 45711 | NRMVLASTTAKAMEQMAGSS | 20 | M1 | 0.29 | 0.94 | 0.86 |
| 7 | 5402 | AVKGVGTMVMELIRMIKRGINDRN | 24 | NP | 0.14 | 0.39 | 0.71 |
| 10 | 67496 | TYVLSIIPSGPLKAEIAQRL | 20 | M1 | 0.50 | 0.29 | 0.71 |
| 11 | 35591 | LELRSRYWAIRTRSGGNTNQQRAS | 24 | NP | 0.08 | 0.68 | 0.57 |
| 12 | 128060 | MSLLTEVETYVLSIIPSGPL | 20 | M1 | 0.29 | 0.39 | 1.00 |
| 14 | 41785 | MITQFESLKLYRDSL | 15 | NS2 | 0.03 | 0.55 | 0.86 |
| 15 | 59546 | SLVGIDPFKLLQNSQVYSLIRP | 22 | NP | 0.16 | 0.32 | 0.86 |
| 16 | 97655 | SPGMMMGMFNMLSTV | 15 | PB1 | 0.07 | 0.94 | 0.71 |
| 18 | 70712 | VRESRNPGNAEIEDLIFLARS | 21 | NP | 0.05 | 0.68 | 0.43 |
| 19 | 54595 | RLIQNSLTIERMVLSAFDERRNK | 23 | NP | 0.08 | 0.26 | 1.00 |
| 20 | 97418 | KGILGFVFTLTVPSE | 15 | M1 | 0.07 | 0.94 | 1.00 |
| 22 | 14070 | ERRNKYLEEHPSAGKDPKKT | 20 | NP | 0.10 | 0.48 | 0.29 |
| 24 | 69642 | VLMEWLKTRPILSPLTKGIL | 20 | M1 | 0.42 | 0.06 | 0.86 |
| 25 | 97498 | MITQFESLKIYRDSL | 15 | NS2 | 0.03 | 0.10 | 0.71 |
| 26 | 97489 | MAFLEESHPGIFENS | 15 | PB1 | 0.04 | 0.77 | 0.57 |
| 27 | 97487 | LVWMACHSAAFEDLR | 15 | NP | 0.04 | 0.68 | 0.57 |
| 29 | 39239 | LRVLSFIRGTKVSPRGKLSTRG | 22 | NP | 0.22 | 0.06 | 0.86 |
| 30 | 97502 | MRILVRGNSPAFNYN | 15 | PB2 | 0.02 | 0.13 | 0.43 |
| 31 | 36863 | LKGKFQTAAQRAMMDQVRES | 20 | NP | 0.01 | 0.74 | 0.86 |
| 33 | 97347 | GKWVRELILYDKEEIRRI | 18 | NP | 0.01 | 0.23 | 1.00 |
