# Supplementary material for: Augmented CD4+ T-cell and humoral responses after repeated annual influenza vaccination with the same vaccine component A/H1N1pdm09 over 5 years
Source: NPJ Vaccines. 2018 Aug 14;3:37. doi: 10.1038/s41541-018-0069-1 (PMC6092382; doi:10.1038/s41541-018-0069-1)
Supplement: Supplementary file 2 — Clinical trial protocol for 5 year follow up [file 41541_2018_69_MOESM2_ESM.pdf]

PROTOCOL

Safety and Immunogenicity studies of pandemic influenza (H1N1) 2009 vaccine in Bergen

Identifying number H1N1VAC-2009, Version 1
EUDRACT number 2009-016456-43

Prof. Rebecca Jane Cox

Influenza Centre

The Gade Institute

Laboratory Building, 5th Floor

University of Bergen

N-5021 Bergen

Norway

Tel: +47 55 97 46 68

Fax: +47 55 97 46 89

E-post: rebecca.cox@gades.uib.no

2nd August 2015

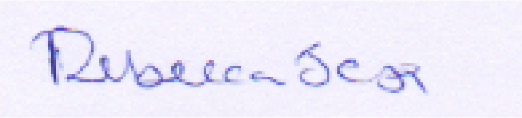

Table of contents

Table of contents **2**

List of tables and figures **5**

List of abbreviations **6**

1. General administrative information **7**

1. Name and address of sponsor **7**
2. Name and title of person authorized to sign protocol **7**
3. Name, title, address and telephone number of sponsors medical expert **7**
4. Name and title of investigators responsible conducting trial **8**
5. Name, title, address and telephone number of qualified physician **8**
6. Name and address of collaborating laboratories **9**
1. Name and description of investigational medicinal product **10**
2. A summary of findings from non clinical studies that potentially have clinical significance and from clinical trials that are relevant to the trial **10**
3. Summary of the known and potential risks and benefits, if any to human subjects **10**
4. Description of and justification for the route of administration, dosage, dosage regimen and treatment period **11**
5. Statement that trial will be conducted in compliance with the protocol, GCP and the applicable regulatory requirements **12**
6. Description of population to be studied **12**
Inclusion criteria 12
Exclusion criteria 12
7. References to literature and data that are relevant to the trial and that provide background for the trial **13**
Background 13
Antigenic shift and drift 13
Influenza Vaccines 14
Safety of inactivated influenza vaccine 15
Kinetics of the immune response to inactivated influenza vaccine 16
European Union (EU) requirements for influenza vaccines 20
Pandemic influenza vaccines 21

4. TRIAL OBJECTIVES AND PURPOSE **23**

5 TRIAL DESIGN **23**

1. A specific statement of the primary endpoints and secondary endpoints to be measured by the trial **23**
A description of the type/design of trial to be conducted 24
2. A description of measure taken to avoid/minimize bias **24**
3. A description of the trial treatment and dosage and dosage regimen of the investigational medicinal product. **24**
Precautions for Use **26**
4. The expected duration of subject participation and a description of the sequence and duration of all trial periods. **26**
STUDY PROCEDURE FOR SUBJECTS IN THE IMMUNOGENICITY AND KINETIC STUDIES 29
First visit: Day 0: First vaccination 29

Second visit: Day 7 (±2 day) post 1st vaccination 30
Third visit: Day 14 (±2 days) post 1st vaccination 30
Fourth visit: Day 21 (±4 days): Second Vaccination 30
Fifth visit: Day 7 (±2 day) post 2nd vaccination ..... **Error! Bookmark not defined.**
Sixth visit: Day 14 (±2 day) post 2nd vaccination ..... **Error! Bookmark not defined.**
Seventh visit: Day 21 (±4 days) post 2nd vaccination ..... **Error! Bookmark not defined.**
Eighth visit: 6 months (±31 days) post 2nd vaccination 30
Ninth visit: 12 months (± 31 days) post 2nd vaccination 31
Tenth visit: 18 months (± 31 days) post 2nd vaccination 31
Eleventh visit: 12 months (± 31 days) post 2nd vaccination 31
Laboratory Analysis for the immunogenicity study 31
5. A description of the stopping rules for individuals, parts of trial and entire trial 32
6. Maintenance of trial treatment codes and procedures for breaking the codes. 32
7. The identification of any data to be recorded directly in CRFs and to be considered to be source data 32

6 Selection and Withdrawal of subjects 33
1. Subjects inclusion criteria 33
Inclusion criteria 33
2. Subjects exclusion criteria 33
3. Subjects withdrawal criteria 34
When and how to withdraw subjects from the trial 34
The type and timing of data to be collected for withdrawn subjects 34
Whether and how subjects are to be replaced 34
The follow-up for subjects withdrawn from treatment 34

7. Treatment of Subjects 34
1. Treatment to be administered including name of all products, the doses, the dosing schedule, the route of administration and the treatment periods including follow-up periods for the subjects for each product treatment group. 34
2. Medication permitted (including rescue medication) and not permitted before and /or during trial 35
3. Procedures for monitoring subject compliance 35

8 Assessment of Efficacy 35
1. Specification of efficacy parameters 35
2. Methods and timing for assessing recording and analyzing efficacy parameters 36
Deviation from protocol 37
Protocol amendments 37

9 Assessment of Safety 38
a. Adverse events (AE) 38
Definitions 38
Surveillance, reporting, and documentation of adverse events 39
Documentation of solicited adverse events 39
Documentation of adverse events 41
Reporting of serious adverse events 41
Causality of adverse events 42
Severity of adverse events 43
Follow-up of ongoing adverse events and assessment of outcome 43
Follow-up of serious adverse events 44
Treatment of adverse events 44

10. Statistics 44
1. A description of the statistical methods to be employed 44
2. The number of subjects planned to be enrolled and reason for choice of sample size 45

Deviation from protocol and procedures for accounting for missing data........45

11. Direct access to source data/documentation 45

12 Quality Control and Quality Assurance 46

13. Ethics 46

Good Clinical Practice........47
Regulatory authority approval........47
Ethics committee approval........47
Patient informed consent........47
Removal/withdrawal of subjects from treatment or assessment........47

14 Data handling and record keeping 48

Storage and retention of study documentation........48

15 Financing and Insurance 48

16 Publication Policy 49

17 Supplements 49

18 References 50

List of tables and figures

Figure 1.1 18
Figure 1.2 19
Table 5.1 27
Figure 5.1 28

List of abbreviations

ASC	antibody-secreting cells
BSL	Biological Safety Level
CHMP	Committee for Medicinal Products for Human Use
CRF	case report form
EDTA	ethylenediaminetetraacetic acid
ELISA	enzyme-linked immunosorbent assay
ELISPOT	enzyme linked immunospot assay
EU	European Union
FACS	Fluorescence activated cell sorting
GCP	Good Clinical Practice
GMP	Good Manufacturing Practice
HA	haemagglutinin
HPA	Health Protection Agency
HI	haemagglutination inhibition
ICH	International Conference on Harmonisation
IM	intramuscular
MCV	mean cell volume
NA	neuraminidase
NIBRG-14	Depathogenised A/Vienam/1194/2004 (H5N1)
NIBSC	National Institute for Biological Standards and Control
NSD	Norsk samfunnsvitenskapelig datatjeneste
PR8	A/Puerto Rico/8/34 (H1N1) (PR8)
SAE	Serious Adverse Event
SUSAR	Suspected Unexpected Serious Adverse Reaction
SOP	standard operating procedure
SRH	single radial haemolysis
UK	United Kingdom
USA	United States of America
UNISI	University of Sienna

1. General administrative information

1. Name and address of sponsor

University of Bergen

Dr. Ph.D. Rebecca Jane Cox
Head Influenza Centre
Influenza Centre
Section for Microbiology and Immunology,
The Gade Institute,
Laboratory Building, 5th Floor,
N-5021 Bergen,
Norway

2. Name and title of person authorized to sign protocol

Dr. Ph.D. Rebecca Jane Cox

3. Name, title, address and telephone number of sponsors medical expert

Dr. cand, med. Per Espen Akselsen
Consultant Centre for Infection Control
Haukeland University Hospital,
N-5021 Bergen
Norway
Tel: +47 55 97 53 80
Fax: +47 55 97 58 13
E-mail: per.akselsen@helse-bergen.no

Professor, dr. med. Haakon Sjursen
Consultant Infectious Diseases Unit,
Haukeland University Hospital,
N-5021 Bergen
Norway
Tel: +47 55 97 29 24
Fax: +47 55 97 29 50
E-mail: haakon.sjursen@helse-bergen.no

Professor, dr. med. Nina Langeland
Head Infectious Diseases Unit,
Haukeland University Hospital,
N-5021 Bergen,

Norway
Tel: +47 55 97 29 61
Fax: +47 55 97 29 50
E-mail: nina.langeland@helse-bergen.no

4. Name and title of investigators responsible conducting trial

Principal Investigator:

Dr., Ph.D. Rebecca Jane Cox

Clinical investigators:

Dr. cand. med. Per Espen Akselsen
Professor, dr. med. Haakon Sjursen

Professor, dr. med. Nina Langeland

5. Name, title, address and telephone number of qualified physician

Dr. cand, med. Per Espen Akselsen
Consultant Centre for Infection Prevention
Haukeland University Hospital,
N-5021 Bergen
Norway
Tel: +47 55 97 53 80
Fax: +47 55 97 58 13
E-mail: per.akselsen@helse-bergen.no

Professor, dr. med. Haakon Sjursen
Consultant Infectious Diseases Unit,
Haukeland University Hospital,
N-5021 Bergen
Norway
Tel: +47 55 97 29 24
Fax: +47 55 97 29 50
E-mail: haakon.sjursen@helse-bergen.no

Professor, dr. med. Nina Langeland
Head Infectious Diseases Unit,
Haukeland University Hospital,
N-5021 Bergen,
Norway
Tel: +47 55 97 29 61

Fax: +47 55 97 29 50
E-mail: nina.langeland@helse-bergen.no

6. Name and address of collaborating laboratories

Drs. Rebecca Cox and Abdullah Madhun
Influenza Centre
University of Bergen,
The Gade Institute,
Laboratory Building, 5th Floor,
N-5021 Bergen,
Norway
Tel: +47 55 97 46 68/67
Fax: +47 55 97 46 89
E-mail: rebecca.cox@gades.uib.no, abdullah.madhun@gades.uib.no

Dr. John Wood
Division of Virology,
National Institute for Biological Standards and Control (NIBSC)
Blanche Lane,
South Mimms,
Potters Bar,
Hertfordshire
EN6 3QG,
UK.
Tel: +44 1707 641309
Fax. +44 1707 646730,
E-mail: jwood@nibsc.ac.uk

Professor Maria Zambon
Head of Respiratory Unit, Health Protection Agency (HPA) Colindale,
Enteric, Respiratory & Neurological Virus Laboratory, 61 Colindale Avenue,
LondonNW9 5HT, UK.
Tel: +44 20 8200 4400 Ext 6269,
Fax: +44 20 8205 8195
E-mail: maria.zambon@hpa.org.uk

Prof. Emanuele Montomoli
Dept. of Physiopathology Experimental Medicine and Public Health
University of Siena - Via Aldo Moro 3 - 53100 Siena - Italy -
Phone: +39 0577234134
Fax: +39 0577234090
E mail: montomoli@unisi.it

Assistant Professor Florian Krammer
Department of Microbiology, Icahn School of Medicine at Mount Sinai
One Gustave L. Levy Place, Box 1124, New York, NY 10029 USA
Tel: +1 212-241-8166
E mail: florian.krammer@mssm.edu

2. Background Information

1. Name and description of investigational medicinal product

The influenza virus strain used for the production of the drug substance is an A/California/7/2009-like virus (H1N1). For production, the virus is grown in the allantoic cavity of embryonated hens' eggs from healthy flocks. The working virus seed lots used have been tested for the absence of bacterial, fungal, and mycoplasma contamination. Pandemrix is a split virion inactivated influenza vaccine. The final formulation contains 3.75 µg haemagglutinin (HA) of A/California/7/2009-like virus (H1N1) per 0.5 ml dose adjuvanted by the proprietary adjuvant AS03, which is composed of squalene (10.69 milligrams), DL-α-tocopherol (11.86 milligrams) and polysorbate 8 (4.86 milligrams). The vaccine will have been licensed by the European Medicines Agency (EMEA) before commencement of mass vaccination at Haukeland University Hospital.

Interactions with other medications: No interaction studies have been conducted. There are no data on co-administration of Pandemrix with other vaccines.

2. A summary of findings from non clinical studies that potentially have clinical significance and from clinical trials that are relevant to the trial

Pandemrix was granted Marketing Authorisations in the EU in May 2008, in subjects' aged 18-60. On 29 May 2009 the Committee for Medicinal Products for Human Use (CHMP) accepted that the age range be extended to >60 years old. The Pandemrix vaccine containing a pandemic A/H5N1 candidate has been found safe and well tolerated in approximately 5000 subjects in these age groups. Pandemrix containing A/H1N1 has undergone initial clinical trials before being licensed by EMEA.

3. Summary of the known and potential risks and benefits, if any to human subjects

Pandemrix was granted Marketing Authorisations in the EU in May 2008, in subjects' aged 18-60. On 29 May 2009 the Committee for Medicinal Products for Human Use (CHMP) accepted that the age range be extended to >60 years old. The vaccine contains a pandemic A/H5N1 candidate has been found safe and well tolerated in approximately 5000 subjects in these age groups.

Pandemrix has been found to be commonly or very commonly associated with a range of local and systemic adverse reactions but these are not often of severe intensity. More information on the CHMP evaluation can be found in EMEA/285631/2008

4. Description of and justification for the route of administration, dosage, dosage regimen and treatment period

Influenza vaccines are routinely administered intramuscularly (IM) or deep subcutaneously. In immunologically naïve subjects (e.g. children), two doses of vaccine are given at 4-week intervals. Influenza vaccines are standardised according to the concentration of HA, and 15µg of each strain is used in the seasonal influenza vaccines. Pandemrix was initially licensed by CHMP for two doses administered at 3 week intervals on 29th September 2009 and then on 20th November for a single dose of vaccine of 3.75 µg HA adjuvanted AS03.

Haukeland University Hospital has approximately 8000 employees many of whom will be in the front line to receive the pandemic A/H1N1 vaccine. We propose to conduct a safety and immunogenicity study in these subjects as detailed below:

- Examine adverse events of all employees after vaccination using adverse events form
- Examine the immunogenicity of the vaccine by collecting blood samples at days 0, 21 & 42 after vaccination (up to 500 people)
- Provide a detailed time course of the immune response to vaccination with a novel influenza H1N1 virus including the kinetics of the T-cell, B-cell and antibody responses in up to 60 volunteers
- Investigate the long-lasting immunity induced by the vaccine
- Examine the ability of the vaccine to induce cross-reactive immunity to H1N1 strains.
- Examine the immune response after seasonal influenza vaccination

All employees will receive one dose of H1N1 influenza vaccine by intramuscular injection into the deltoid muscle.

The pandemic A//H1N1 influenza vaccine is supplied in multidose vials and the vaccine and adjuvant are mixed according to the manufacturers instructions. The appropriate volume of vaccine is drawn up in a syringe as a suspension for injection . The intramuscular injection will be given into the centre of the deltoid muscle between the shoulder and axilla and between the back and front of the arm. Before injection, the syringe plunger will be drawn back in order to check

that the injection is not administered intravascularly. After vaccination, all subjects must remain under observation by the study staff for at least 30 minutes.

5. Statement that trial will be conducted in compliance with the protocol, GCP and the applicable regulatory requirements

This study is compliant with all aspects of ICH / Good Clinical Practice and local regulations.

6. Description of population to be studied

Subjects (aged 19-70 years) will be recruited primarily from employees or students at Haukeland University Hospital or the University of Bergen. Subjects included in the safety analyse of adverse reactions will be informed about the study prior to vaccination by subject information letter. Subjects who will be asked to provide blood samples will be briefly informed of the nature of the study in a lecture and by the subject information letter. They will be enrolled during subsequent one to one interviews when they will receive a detailed explanation of the study protocol. If the subject meets the inclusion and does not meet the exclusion criteria and signs the informed consent form, they will be enrolled in the trial and allocated a unique subject identification number. Subjects will be considered eligible to enter the study provided they satisfy the following criteria:

Inclusion criteria

- Signed informed consent
- Subjects able to understand and comply with the study protocol and complete the Adverse Event Form
- Subjects able to attend the scheduled visits for the kinetic study

Exclusion criteria

- Persons with a history of anaphylaxis or serious reactions to any vaccine
- Person with known hypersensitivity to any of the vaccine components
- Persons who have had a temperature >38°C during the previous 72 hours
- Persons who have had an acute respiratory infection during the last 7 days

- Suspected non-compliance

7. References to literature and data that are relevant to the trial and that provide background for the trial

Background

Influenza virus is a globally important respiratory pathogen, which annually causes high levels of morbidity and mortality. Three types of influenza virus have been identified (A, B, and C), but only influenza A and B cause overt disease and result in regular outbreaks or epidemics. Influenza A viruses are further subdivided into subtypes based on the surface antigens, haemagglutinin and neuraminidase. Today, 16 subtypes of HA (H1−H16) and 9 subtypes of NA (N1−N9) have been found in influenza A viruses, and all the subtypes are found in aquatic birds.

Antigenic shift and drift

Influenza virus in man continuously undergoes antigenic changes in the surface glycoproteins (HA and NA) to escape the host's acquired immunity, so-called antigenic drift. Antigenic drift is responsible for the inter-pandemic outbreaks of influenza and consequently the vaccine has to be annually updated.

Aquatic birds are the natural reservoir for influenza A viruses, harbouring all known subtypes, and constitute a risk for the introduction of new influenza A subtypes to man. The appearance of such an influenza A virus (with novel surface glycoproteins HA and/or NA) able to infect and spread in an immunologically naïve human population is designated as antigenic shift. This occurs at infrequent and unpredictable intervals resulting in a pandemic with the potential to cause exceptionally high levels of global morbidity and mortality. There were three pandemics during in the last century (1918, 1957 and 1968) and although each was associated with worldwide spread of virus, the highest death toll was seen in 1918-19, when 20-40 million people died, many of whom were young healthy adults. Two of the antigenic shifts, which occurred in the 20th century, were probably due to reassortment between human and avian viruses possibly via an intermediate host (most likely pigs) serving as a mixing vessel. The sudden emergence and the rapid global spread of swine-origin influenza A (H1N1) surprised many. The World Health Organization declared an influenza pandemic (Phase 6) on June 11 after nearly 30,000 confirmed cases were reported in 74 countries (1).

Preceding and following the transition to Phase 6, the new virus was genetically and antigenically characterised (2) (3, 4), (5), (6). There was a general consensus about the origin of the novel H1N1 virus, being the result of multiple reassortments steps between human H3N2, North American avian virus, and H1 from swine viruses from the North American and Eurasian lineages. The gestation period for this new H1N1 virus apparently started in the late 1990's and culminated in swine at some undefined recent time-point prior to the emergence of the final virus in man. Also, the antigenic properties clearly indicated that the HA of the novel H1N1 strain was only distantly related to current seasonal H1N1 viruses (6). This virus is easily transmissible to humans and generally causes mild disease, although severe illness (respiratory distress) and fatalities have been reported, mostly so during the first weeks in Mexico (2). The worldwide spread of the novel influenza A (H1N1) and the continuous sporadic zoonotic cases of avian H5N1 increase the risk of the reassortment between the two viruses and therefore constitute an additional global threat. Inactivated vaccines against influenza virus was first made during the 1940s, initially aimed at military personnel, but later also targeted for the civilian population, particularly the elderly and those at risk of serious illness and death. Our best option to mitigate the health and societal consequences of a pandemic is to have access to an efficacious vaccine in a timely manner (7). Vaccination is the best option by which spread of a pandemic virus could be slowed down or halted and severity of disease reduced.

Influenza Vaccines

While antivirals play a role in disease control, vaccination remains the most effective prophylactic measure to prevent morbidity and mortality (reviewed by (8)). Two types of influenza vaccine are available; inactivated vaccine delivered deep subcutaneously or intramuscularly and live attenuated vaccine administered intranasally. Inactivated influenza vaccines have been licensed for many decades and are 60-80% effective in preventing disease with homologous or closely related strains. In some cases, vaccination may not prevent influenza illness, however it reduces the severity and duration of the illness. Inactivated influenza vaccines are used routinely every year and they are considered to be safe. Inactivated vaccines are available in whole, split (chemically disrupted), and subunit (purified surface glycoproteins) formulations.

Current inactivated vaccines are produced by propagation in embryonated hens' eggs. The allantoic fluid is harvested, and the virus is concentrated and highly purified, then inactivated with formaldehyde or beta-propiolactone. The availability of embryonated hens' eggs is a limiting factor in vaccine production and the global manufacturing capability is not expected to meet

pandemic vaccine requirements, thus it is important to develop dose-sparing strategies by using effective adjuvants. The use of reverse genetics technology can save a considerable amount of time in production of a pandemic vaccine. Traditionally, influenza vaccines have been made by choosing a virus isolate which closely matches circulating strains, and introducing its surface glycoprotein genes (HA and NA) into the genetic background of the PR8 virus. Seed viruses are produced by co-infection of the two "parent" viruses, and screening for the progeny of interest. This screening from a possible 256 progeny genotypes is time consuming, and may be unsuccessful. The use of reverse genetics allows viruses to be constructed with a prescribed combination of genes, following combination of plasmid DNAs encoding separate RNA segments. Genetic manipulation allows engineering of the DNAs to create viruses altered in specific genes for example attenuation of highly pathogenic avian viruses to create a depathogenised virus, which can be used as a vaccine strain (reviewed in (9)). A rapid response to a pandemic threat by production of a safe, effective vaccine is one of the most important objectives in pandemic control. The potential to save valuable time in preparing a pandemic influenza vaccine and provide essential information on the optimal use of the vaccine could have an important impact on the public health. Should a pandemic occur today, a safe, immunogenic and appropriately formulated vaccine is urgently needed which could be rapidly prepared in sufficient quantities. Current seasonal influenza vaccines contain 15 μg HA from each of the three seasonal strains. In contrast, very high antigen doses (up to 90 μg HA) are required for non-adjuvanted split virus or recombinant pandemic H5 vaccines to elicit an antibody response sufficient to meet the CHMP criteria (10, 11). This underlines the need for effective adjuvants to enhance the immune response to split or subunit pandemic influenza vaccines. The best candidate vaccine formulation currently available is an adjuvanted inactivated virus vaccine (reviewed in (12); (13); (14)).

Safety of inactivated influenza vaccine

Millions of doses of inactivated egg-grown influenza vaccine are administered each year, and the vaccine has excellent safety and tolerance profiles (15); (16), with very low numbers of adverse reactions reported. Mild local reactions consisting of tenderness and redness at the injection site are often observed after inactivated influenza vaccination in more than 50% of healthy adults, although these are mild and rarely interferes with normal activities (17). Although systemic reactions such as fever, malaise, myalgia and headache are recorded, controlled trials have not been able to establish a difference between systemic symptoms associated with receipt of vaccine

and those associated with receipt of placebo (17). Immediate (allergic) reactions or hypersensitivity to influenza vaccines occur extremely rarely (in Norway, the incidence of anaphylactic shock for all types of vaccine is estimated to be less than 5 cases per 800,000 (18). There have been reports of an association between Guillain Barré Syndrome and influenza vaccine, but it is estimated that the overall risk is only 1 case per million persons immunised (19)). The inactivated influenza vaccine for this trial is an adjuvanted split virus vaccine so the above considerations apply. The mock up adjuvanted avian A/H5N1 vaccine has undergone clinical trial in approximately 5000 volunteers. In these subjects the incidence of adverse reactions is higher than in non adjuvanted vaccine recipients, particularly for local reactions, however the incidence of server reactions was low (EMEA/285631/2008).

Kinetics of the immune response to inactivated influenza vaccine

Antibodies to the surface glycoproteins, HA and NA, of seasonal influenza strains are associated with resistance to infection, whereas antibodies to the conserved internal antigens, M and NP, are not protective (20). The cytotoxic T-cell response is mainly directed against the M and NP proteins and is important for clearance of the virus and recovery from illness.

Vaccination with inactivated vaccine results in both local and systemic responses. The serum antibody response increases as early as 2-6 days after influenza vaccination in primed subjects (21) and peaks at approximately 2 weeks after vaccination when 90% of vaccinees have protective antibody titres (21); (17). The serum antibody response then wanes over time and is generally two-fold lower 6 months after vaccination (22). A rapid increase in local secretory IgA antibody in the oral fluid is also observed after parenteral inactivated influenza vaccination but this antibody response only remains elevated for 3 weeks after vaccination (23, 24). Influenza-specific antibody-secreting cells (ASC) appeared earlier, at approximately 7 days post-vaccination, in the blood than serum antibody response (21) and consisted predominantly of IgG and IgA. In young children (2 to 3 years old), previous natural priming by influenza infection was essential to mount strong antibody and antibody secreting cell responses in the peripheral blood (25, 26).

The quality and magnitude of the immune response after influenza vaccine

Innate immune system

The immune system is commonly divided into innate and adaptive arms. Innate immunity is characterized by an immediate response against an invading pathogen as a response to pathogen associated molecular patterns (PAMP) common for an array of microorganisms (27). Professional

antigen presenting cells (e.g. dendritic cells) are usually the first immune cells to encounter pathogens, and are therefore very important for presentation of antigens to the adaptive immune system.

Adaptive immune system

In contrast to innate immunity, an adaptive immune response is slower, but highly pathogen-specific. Adaptive immunity is also characterized by memory, and can give a rapid and strong response after re-encountering pathogens. The main cell types of the adaptive immune system are B and T lymphocytes. B cells produce antibody capable of neutralizing and opsonizing pathogens. T cells are divided into T helper (Th) $CD4^+$ cells and T cytotoxic (CTL) $CD8^+$ cells. The CTL exert their effector function on virally infected cells by releasing perforin and granzyme B (GranB), which leads to cell death (reviewed in (28)). Th cells orchestrate the immune response by secreting cytokines and can be divided into two main subsets namely Th1 and Th2 based on the cytokines they produce which influence the proliferation and differentiation of other immune cells. In man, IL-2, IFN-γ and TNF-α characterize a Th1 response, which enhance the cell-mediated immunity and cytotoxic response. On the other hand, a Th2 profile is associated with IL-4, IL5 and IL-10 secretion, which promote B cell induction and humoral immunity.

Antibodies to the surface glycoproteins, HA and NA, of seasonal influenza strains are associated with resistance to infection, whereas antibodies to the conserved internal antigens, M and NP, are not protective (20). The cytotoxic T-cell response is mainly directed against the M and NP proteins and is important for clearance of the virus and recovery from illness.

Vaccination with inactivated vaccine results in both local and systemic responses. The serum antibody response increases as early as 2-6 days after influenza vaccination in primed subjects (21) and peaks at approximately 2 weeks after vaccination when 90% of vaccinees have protective antibody titres (21); (17). The serum antibody response then wanes over time and is generally two-fold lower 6 months after vaccination (22). Influenza-specific antibody-secreting cells (ASC) appeared earlier, at approximately 7 days post seasonal influenza vaccination, in the blood than serum antibody response (21) and consisted predominantly of IgG and IgA. In young children (2 to 3 years old), previous natural priming by influenza infection was essential to mount strong antibody and antibody secreting cell responses in the peripheral blood (25, 26). On the other hand, two doses of pandemic H5N1 induced a peak antibody response 3 weeks (measured by HI and ELISA) after the first vaccine dose and 2 weeks after the second dose (unpublished data, Fig 1.1).

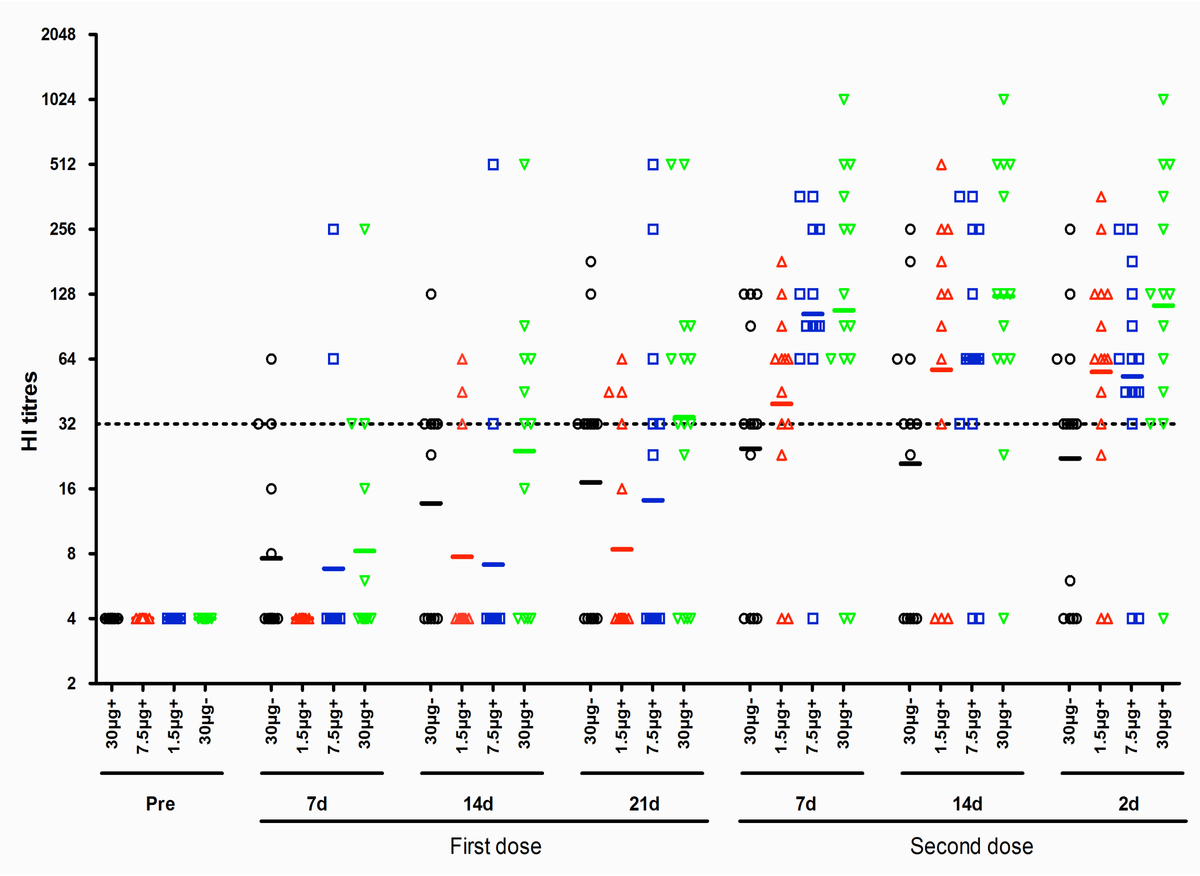

Figure 1.1. The kinetics of serum haemagglutination inhibition (HI) antibody response after vaccination measured by the modified HI test.
Sixty health adults were intramuscularly vaccinated with 2 doses of virosomal influenza A H5N1 vaccine with or without ISCOM-adjuvant. Each symbol represents the HI titre for one individual participant, with the geometric mean titre (GMT). The vaccine dose and presence (+) or absence (−) of ISCOM adjuvant and the sampling day after vaccination are shown on the *x*-axis.

A peak ASC response was observed 7 days after the first dose and 4-6 days after the second vaccine dose. In a clinical trial of a cell derived pandemic H7N1 vaccine, subjects who elicited an antibody response had higher number of antibody secreting cells and an association with IL-2 production (29)

Recent animal studies have shown that pandemic vaccines may provide protection despite a low antibody response suggesting the need for better understanding of vaccine-induced immunity and correlates of protection to pandemic influenza viruses (30, 31). However, there is growing evidence, that both $CD4^+$ Th cells and $CD8^+$ CTL may not only play an important role in controlling viral infection, but also may reduce the severity of disease and decrease mortality (32, 33). Therefore, investigating the quality of the T cell response after vaccination is crucial for understanding vaccine-induced immunity. A novel approach using flow cytometry to study multifunctional Th1 (e.g. TNF-α^+IFN-γ^+IL-2^+) cells was recently used to evaluate the immune response after vaccination in different infection models and induction of this subset of T cells was

associated with the protective efficacy of vaccine ((34)and reviewed in (35)). The results provided evidence that the quality "multifunctionality" of the cytokine producing T cell induced after vaccination is a better indicator of a protective T-cell response than the magnitude of the response. Our preclinical studies and clinical trial showed that ISCOM-adjuvanted pandemic H5N1 vaccine induced high frequencies of TNF-α^+IFN-γ^+IL-2^+ T cells (submitted manuscript and unpublished data, Fig 1.2). Additionally, these T cells showed high cross-reactivity to other H5N1 clades in both human and mouse. The use of multifunctional T cell response as a potential surrogate marker of protection against influenza remains to be established.

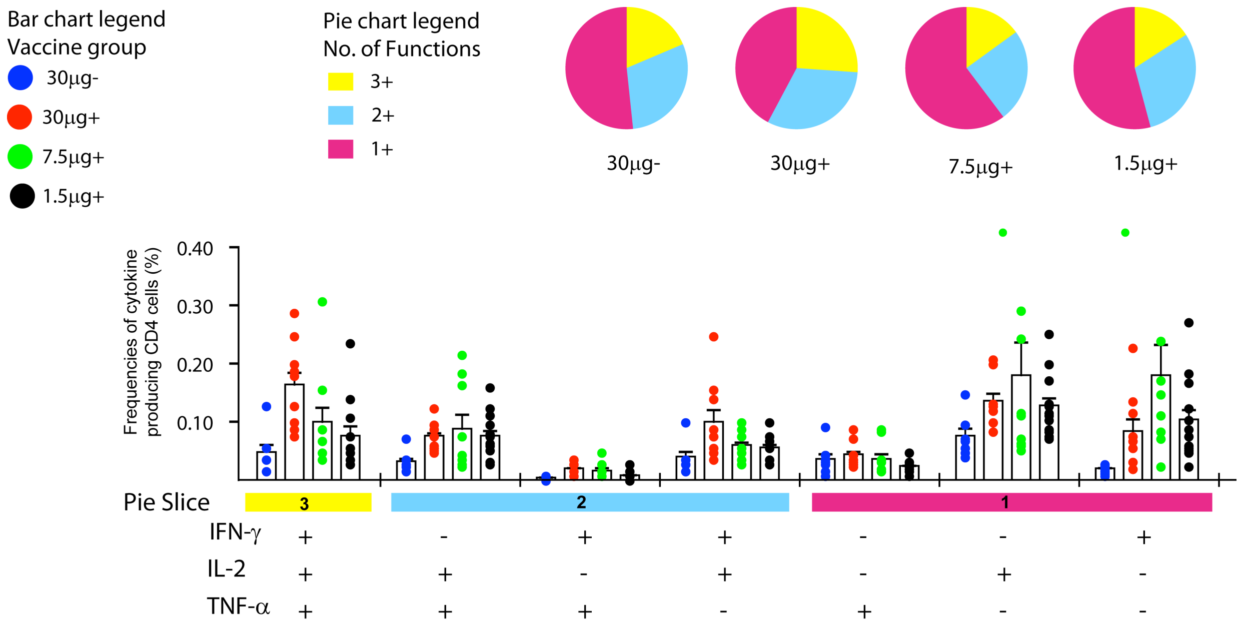

Figure 1.2: The multifunctional influenza-specific cytokine secreting Th1 CD4+ response induced in blood after influenza vaccination.
Sixty health adults were intramuscularly vaccinated with virosomal influenza A H5N1 vaccine with (+) or without (-) ISCOM-adjuvant. Peripheral blood lymphocytes were activated in vitro 21 days after second vaccine dose and were intracellularly stained and analysed by flow cytometry. The subjects were divided into intramuscular non-adjuvanted (30µg-) and adjuvanted (1.5µg, 7.5µg and 30µg) groups. The data show the frequencies of cells expressing each of the seven possible combinations of IFN-γ, IL-2 and TNF-α for each individual and lines represent the mean ± SEM. The pie charts show the fraction of the total response comprising cells expressing all three cytokines (3+), any two cytokines (2+), or any one cytokine (1+).

Very few reports have addressed the long-lasting immunity and memory response after pandemic influenza vaccines (36). Most of the published data evaluated the response in the weeks after vaccination, which may underestimate the long-term protection against influenza viruses. Current seasonal influenza vaccines are known to induce antibodies which last 6-12 months after

vaccination and therefore, annual vaccination is needed even against an influenza strain that is not updated from the previous season. The optimal vaccine should mount long-lasting immunity and memory responses. Humoral long-lasting immunity can be assessed by measuring influenza-specific antibodies months or years after vaccination using the classical serological assays such as enzyme linked immunosorbant assay (ELISA) and hemagglutination inhibition (HI). Long-lived plasma cells which continuously secret antibodies can be detected in the bone marrow months after vaccination (37). There is evidence that both memory T and B cells are also induced after infection or effective vaccination. Therefore, it will be very important to evaluate the memory response induced after pandemic influenza vaccines. The frequencies of these cells can be measured by antigen or/and polyclonal activation and enzyme linked immunospot (ELISPOT)-based assay as previously described (38).

European Union (EU) requirements for influenza vaccines

The Committee for Medicinal Products for Human Use (CHMP) has defined a set of regulatory criteria influenza vaccines need to meet in order to obtain a marketing license in the European Economic Area (39). The vaccine efficacy is tested by the haemagglutination inhibition (HI) (40{Kendal, 1982 #224), or single radial haemolysis (SRH) assays (41). An HI titre ≥ 40 (or an equivalent SRH > 25mm^2) indicates 50% protective levels of serum antibody against influenza infection in man. The EU requirements for harmonisation of influenza vaccines require annual clinical trials for licensing. The vaccine should fulfil one of the following evaluation criteria for each strain in adults aged 18-60:

1. The number of seroconversions or significant increases in anti-haemagglutinin antibody titre should be > 40%;
2. Mean geometric increase >2.5;
3. The proportion of subjects achieving an HI titre ≥40 or SRH > 25mm^2 should be >70%.

There are no requirements for inactivated vaccines to evoke a cellular immune response. The World Health Organisation (WHO) has prepared a number of recommendations for increased preparedness and research (42). The CHMP require pandemic vaccine to meet all the current evaluation criteria for marketing authorisation. They also recommend measuring neutralising antibodies, and suggest more detailed analysis of the immune response for example detection of antibodies directed against neuraminidase and cell mediated responses (43).

Pandemic influenza vaccines

Parenterally administered inactivated influenza vaccines have been used for many decades and extensive information is available on their quality, safety and efficacy. These vaccines elicit an effective systemic immune response in individuals previously primed by natural infection but much less information is available on the immune response to influenza vaccines containing novel subtypes. During an influenza pandemic, vaccines will be a key intervention step in protecting the population. The type of vaccine, the amount of antigen, the route of administration, the number of doses and the incorporation of an adjuvant, in addition to the immunological status of the recipient, will all influence the immune response.

The use of a single dose regimen with a novel subtype is unlikely to be suitable in an immunologically naïve population, whereas a two dose regime more often results in seroconversion and fulfilment of the CHMP requirements (44)-(45, 46). The current influenza vaccine manufacturing capacity will not be sufficient to meet the global demand for a pandemic vaccine, particularly if two doses of vaccine are required to elicit an adequate antibody response (10, 46-50). Whilst whole virus vaccines have proved more immunogenic in unprimed adults than split or subunit vaccines (46, 51), most vaccine manufacturers are technically unable to convert their current split or subunit vaccine production process to whole virus production. Current seasonal influenza vaccines contain 15 μg HA from each of the three seasonal strains. In contrast, very high antigen doses (up to 90 μg HA) are required for non-adjuvanted split virus or recombinant pandemic H5 vaccines to elicit an antibody response sufficient to meet the CHMP criteria (10, 11).

Aluminium-salt adjuvants are readily available and inexpensive, and have been shown to modestly augment the antibody response after two doses of candidate pandemic influenza vaccines, either split (50) or whole virus formulations (44, 51). However, other trials found that aluminium adjuvant did not significantly enhance the antibody response to candidate H5 vaccines (49, 52, 53). The most promising adjuvants are the proprietary oil-in-water emulsion systems such as MF59, AS03 and AF03, which greatly enhanced homologous and cross reactive antibody responses after H5 vaccination (45, 48, 53-55) even at lower antigen concentrations (3.8 - 7.5 μg HA). There are more than 70 clinical trials of candidate pandemic influenza vaccines, which have been completed or are ongoing (56). To date pandemic influenza vaccines have been reported as safe and well tolerated, with most trials performed in healthy adults. A number of pandemic vaccines have met regulatory approval in Europe, the USA and Australasia including Pandemrix.

Most vaccine manufacturers are now producing pandemic influenza A (H1N1) 2009 vaccine and the first vaccines entered clinical trial in August 2009.
Importantly, the goal of pandemic vaccination is to elicit appropriate immunological effector mechanisms to reduce viral replication and thus viral shedding and provide protection against serious illness and death upon infection by pandemic influenza. Protective efficacy studies using pandemic candidate H5 vaccines in animal models have shown that correlates of resistance to serious illness and death to avian viruses may not be solely reflected by levels of circulating serum antibodies (30, 57-59). These findings raise concern about the use of the CHMP criteria for assessing surrogate correlates of protection to candidate pandemic vaccines and there is therefore a need for more research to allow a better understanding of the immune response after pandemic influenza vaccination in man.

The WHO has provided advice for deployment of pandemic influenza H1N1 vaccine which would ensure the integrity of the health-care system and the country's critical infrastructure; reduce morbidity and mortality; and reduce transmission of the pandemic virus within communities. The Norwegian Government has ordered enough pandemic A/H1N1 vaccine for two doses for the whole population. It is anticipated that the pandemic A/H1N1 vaccine should be licensed in October 2009 and the initial batches will arrive in Norway during October but delivery of all the vaccine will be staggered over time. One of the key groups of workers that will be prioritized for the first rounds of vaccination will be frontline healthcare workers. These workers are at increased risk of infection with influenza H1N1 and of transmitting that infection to susceptible patients. Thus, the potential advantages for healthcare workers are personal protection, protection of patients, and reduction of absenteeism.

There are five key criteria, which need to be addressed in clinical trials of pandemic influenza vaccines in man (60); 1) safety, 2) immunogenicity, 3) kinetics of the response, 4) long lasting immunity and 5) cross reactive immunity to influenza H1N1 strains. Furthermore, there is a clear need for improved understanding of the immune response elicited in human volunteers after vaccination with candidate pandemic vaccines including the quality and repertoire of the antibody response. These five criteria will be addressed in this trial as follows: The safety and tolerability of the H1N1 vaccine will be investigated by completion of a side reactions form detailing the local and systemic adverse reactions. The immunogenicity of the H1N1 vaccine will be assessed through the induction of local and systemic antibody and cellular immune responses. In a pandemic situation, an important aspect is the rapidity of the immune response to the H1N1

vaccine so the detailed kinetics of the immune response will be investigated. The capacity of the vaccine to elicit long lasting immunity and cross reactive immunity to H1N1 viruses will also be evaluated. Furthermore, the quality of the immune response induced by the vaccine will be studied. The study population of healthcare workers is a unique population as Bergen is one of the few places in the world that vaccinated before the peak of the pandemic activity allowing evaluation of the vaccine in an immunologically naïve population (61)

4. TRIAL OBJECTIVES AND PURPOSE

The objectives and purpose of this trial are:

1) Evaluation of the local and systemic safety and tolerability of parenterally administered adjuvanted H1N1 influenza vaccine in all recipients of vaccine at Haukeland University Hospital.

To evaluate the immunogenicity of a pandemic H1N1 influenza vaccine including:

2) Provide a detailed time course of the immune response to vaccination with a novel influenza H1N1 virus including the kinetics of the T-cell, B-cell and antibody responses in up to 60 volunteers
3) Investigate the long-lasting immunity induced by the vaccine
4) Examine the ability of the vaccine to induce cross-reactive immunity to H1N1 strains
5) Evaluate the quality of the immune response induced by the vaccine.
6) Examine the immune response after seasonal influenza vaccination in this study population

5 TRIAL DESIGN

1. A specific statement of the primary endpoints and secondary endpoints to be measured by the trial

The primary endpoints of the trial are the local and systemic adverse events and tolerability of parenterally administered adjuvanted H1N1 influenza vaccine in all volunteers. The secondary

endpoints are the evaluation of the immunogenicity of adjuvanted H1N1 influenza vaccine and the ability of the vaccine to meet the CHMP evaluation criteria in a subset of volunteers.

The immunogenicity of the H1N1 vaccine will be assessed through the induction of specific local and systemic antibody and cellular immune responses, and analyses of the epitopes to which the response is directed. Furthermore, the capacity of the vaccine to elicit cross reactive and long lasting immunity will be evaluated. In a pandemic situation, an important aspect is the rapidity of the immune response to the H1N1 vaccine and therefore the detailed kinetics of the immune response will be investigated.

A description of the type/design of trial to be conducted

The clinical trial will be an open study, all subjects eligible for pandemic vaccination at Haukeland University Hospital will receive one dose of vaccine by intramuscular injection into the deltoid muscle. An adverse events form will be dispensed to all subjects.

In the immunogenicity study, blood and oral fluid samples will be collected at the appropriate days (e.g. 0, 7, 14 and 21) after pH1N1 and seasonal vaccination. This study is an open study and all subjects will receive the Pandemrix vaccine, however, the trial samples will be coded with a unique number. Serological assays will be conducted at The University of Bergen and also blinded by the reference laboratories, National Institute for Biological Standards and Control (NIBSC), Health Protection Agency (HPA) and University of Siena (UNISI). Blood samples will also be collected at 3, 6, 9, 12, 18 and 24 months after pH1N1 and seasonal vaccination. The kinetics of the immune response will be studied at the University of Bergen.

2. A description of measure taken to avoid/minimize bias

The study will be an open study. All trial blood samples will be coded and serological assays to be conducted at NIBSC, HPA and UNISI will be performed blinded.

3. A description of the trial treatment and dosage and dosage regimen of the investigational medicinal product.

Subjects will be immunised by intramuscular injection into the deltoid muscle with the pandemic A/H1N1 influenza according to national guidelines.

During the immunogenicity study up to 23 blood (by venepuncture) and 11 oral fluid samples will be collected at intervals after vaccination. The trial blood and oral fluid wash samples will be used to evaluate the antibody, T and B cellular responses induced after vaccination. The oral fluid sample will be collected using an absorbent pad placed on the gum to absorb the oral fluid for two minutes (Orasure™). Blood and oral fluid samples will be collected at appropriate days 0, 7, 14 and 21 after vaccination to allow investigation of influenza specific cellular and serum antibody responses after pH1N1 and seasonal vaccination, as previously described (21, 23, 25, 62-64). These samples will be allocated a unique subject identification number, and only information on the age; sex and previous influenza vaccination history of each subject will be available to the scientific research team. Serum samples will be analysed at the University of Bergen and also sent to the three reference laboratories in Europe for studying the antibody responses.
Sera will be separated from clotted blood samples and plasma will be separated from CPT herparinised blood, aliquoted and stored at -80 ^{o}C or -20°C for use in the HI, SRH, neutralisation assay, enzyme-linked immunosorbent assay (ELISA), HA protein array and other assays deemed appropriate. Lymphocytes will be separated from CPT herparinised blood samples and used directly in the relevant immunological assays e.g. enzyme linked immunospot assay (ELISPOT) and for evaluation of the B and T cellular responses by e.g. flow cytometry (FACS) and cytokine production by multiplex ELISA and FACS. The recently described novel approach using multifunctional T cells (e.g. TNF-α^{+}IFN-γ^{+}IL-2^{+}) will be used to evaluate the immune response after vaccination. This will allow evaluation of the quality or "multifunctionality" of the cytokine producing T cells induced after vaccination and has been found to be a better indicator of a protective T cell response than the magnitude of each cytokine response. Excess lymphocytes will also be stored in liquid nitrogen for use in relevant immunological assays e.g. *in vitro* activation assays at the University of Bergen.

Preparation and Administration

The vaccine will be administered by the Centre for Infection Prevention as part of Haukeland University Hospitals mass vaccination campaign against pandemic influenza A (H1N1) 2009 according to national guidelines.

Before use, the vaccine will be removed from the refrigerator and placed at room temperature for 5 and 10 minutes. The vaccine and adjuvant will be mixed according to the manufacturers instructions. Prior to injection, it will be gently shaken. The vaccine will be injected intramuscularly into the deltoid region (on the opposite arm to that of the blood sampling). To ensure intramuscular injection, the subject will be asked to relax and the needle will be inserted at the center of the deltoid muscle between the shoulder and axilla and between the back and front of the arm. Before injection, the syringe plunger will be drawn back in order to check that the injection is not administered intravascularly. All vaccinations will be recorded in PANVAK according to National guidelines and instructions from The Norwegian Institute of Public Health. The site and side of injection will be recorded for the subjects involved in the immunogenicity and kinetic studies in the patient information and in the case report form (CRF).

Precautions for Use

Vaccination must not be performed in subjects allergic to any VACCINE component.

4. The expected duration of subject participation and a description of the sequence and duration of all trial periods.

Subjects who are only in the adverse reactions study will be involved in the study for a period of 6 weeks. Subjects involved in the immunogenicity and kinetic studies will be involved in the trial for a period of 48-50 months. The main part of the trial will last 6 weeks, but the subjects will be asked to give trial blood samples at 3, 6, 9, 12, 18 and 24 months after pH11N and seasonal vaccination to examine the longevity of the immune response.

A summary of the proposed study plan for the immunogenicity and kinetic studies is given below in the Table 5.1 and Figure 5.2.

Table 5.1 Summary of the time schedule of the immunogenicity study which with the extension of the trial for another 3 year will be repeated

	Vaccination Day 0	Day 7	Day 14	3 months	9 months	6 months	12 months	18 months	24 months
**Allowable time variation (±days)**		±2	±2	±31	±31	±31	±31	±31	±31
**Informed consent**	✓								
**Fulfilment of inclusion/exclusion criteria**	✓								
**Medical /medication history**	✓								
**Antibody studies**	✓	✓	✓	✓	✓	✓	✓	✓	✓
**T- and B- responses**	✓	✓	✓	✓	✓	✓	✓	✓	✓
**Vaccination**	✓								
**Observation period (30 minutes)**	✓								
**Examine vaccination site (30 minutes)**	✓								
**Collection of Adverse Events**	✓	✓	✓	✓	✓				
**Serious Adverse Events**									

The following blood samples will be collected:
Antibody studies (2 x 8mL clotted blood sample)
T- and B- cellular responses (4 x 8mL CPT™ heparinised)

Figure 5.2.

Flow chart of the immunogenicity study

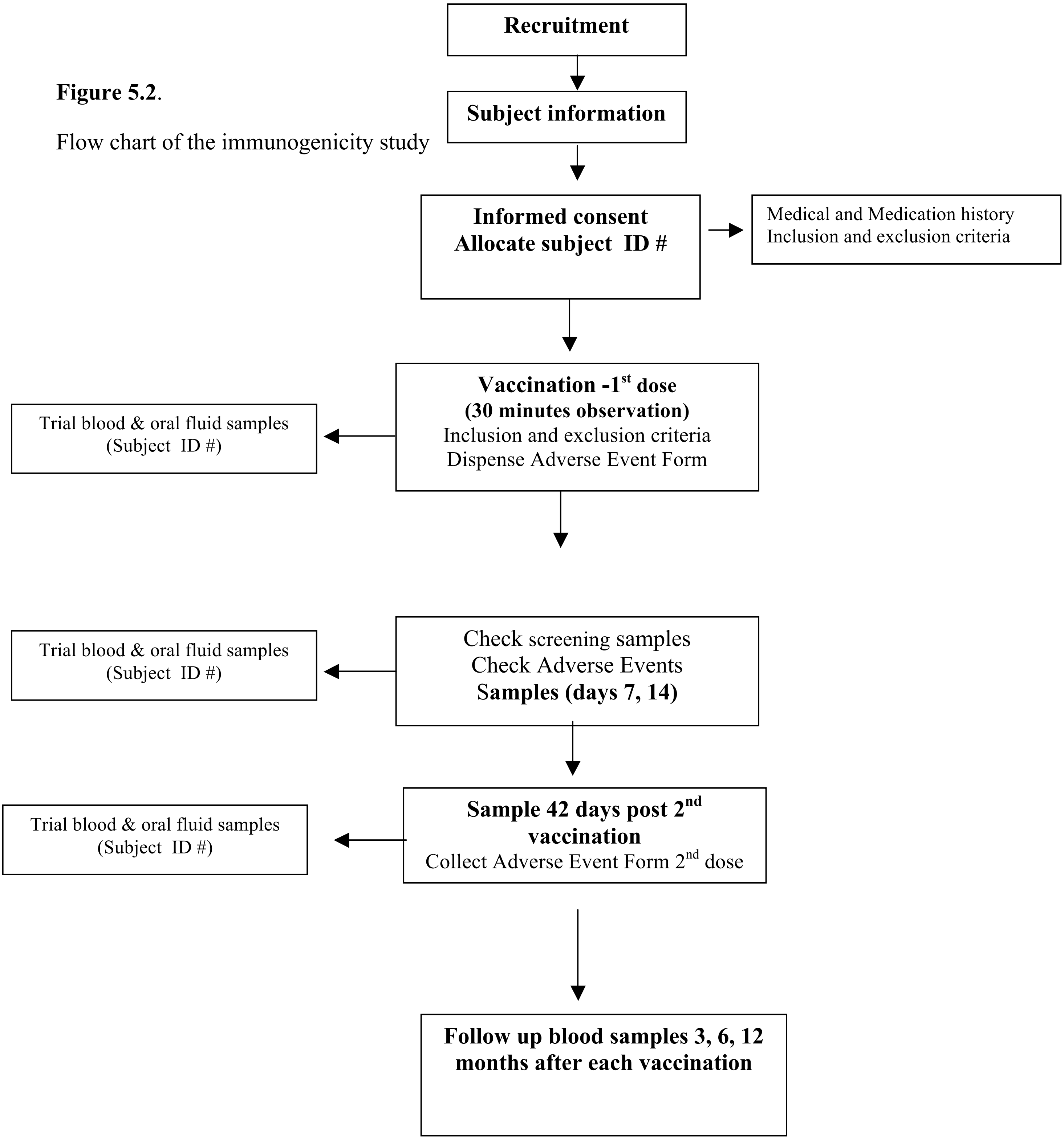

STUDY PROCEDURE FOR SUBJECTS IN THE IMMUNOGENICITY AND KINETIC STUDIES

First visit: Day 0: First vaccination

Subjects will be enrolled following one to one interviews for immunogenicity and kinetic studies providing they meet inclusion and not meeting the exclusion criteria and give fully signed informed consent. The following procedures will then be carried out and recorded in the Case Report Form:

- Record personal details including age, sex, personal number
- Record relevant medical and medication history;
- Record influenza vaccination history;
- Allocate a unique subject identification number;
- Trial blood samples will be collected by venepuncture to measure the antibody, T- and B-cellular and antibody responses.
- An Orasure fluid sample will be collected to measure the local antibody response. The oral fluid sample will be collected using an absorbent pad placed on the gum to absorb the oral fluid for two minutes.
- Subjects meeting the inclusion and not meeting the exclusion criteria will be vaccinated parenterally (into the deltoid muscle of the upper arm (the opposite side to that used for venepuncture)). Vaccination will be recorded in PANVAK according to National guidelines. The side of injection will be recorded in the CRF
- Subjects will be observed for 30 minutes to check for immediate adverse reactions. Emergency equipment (adrenaline, corticosteroids) will be available in the event of a rare anaphylactic reaction to vaccine administration.
- The vaccination site will be examined for local reactions at the end of 30 minutes. These findings and any systemic adverse reactions will be recorded on the appropriate CRF page.
- Dispense the subject’s adverse event forms to the subjects, and give instructions for its completion. The adverse event form must be returned to the site at the Day 21 visit.
- Each subject will be instructed in the evaluation of local and systemic adverse events.
- Schedule the next clinic visit.

Second visit: Day 7 (±2 day) post 1st vaccination

- The adverse event form will be reviewed.
- Trial blood samples will be collected by venepuncture to measure the antibody, T- and B-cellular and antibody responses.
- An Orasure fluid sample will be collected to measure the local antibody response.
- Schedule the next clinic visit.

Third visit: Day 14 (±2 days) post 1st vaccination

- The adverse event form will be reviewed.
- Trial blood and Orasure samples will be collected to measure the antibody, T- and B-cellular and local antibody responses respectively.
- Schedule the next clinic visit.

Fourth visit: Day 21 (±4 days)

During this visit the following will be carried out and recorded in the CRF;

- Collect and discuss Adverse Event Form.
- Obtain interim history, including post injection reactions, adverse events and use of prescription and relief medication
- Trial blood samples will be collected by venepuncture.

Fifth visit: 3 months (±31 days) post 2nd vaccination

- Subjects will be asked to give a trial blood sample.
- An Orasure sample will be collected.

Sixth visit: 6 months (±31 days) post 2nd vaccination

- Subjects will be asked to give a trial blood sample.
- An Orasure sample will be collected.

Seventh visit: 9 months (±31 days) post 2nd vaccination

- Subjects will be asked to give a trial blood sample.
- An Orasure sample will be collected.

Eighth visit: 12 months (± 31 days) post 2nd vaccination

- Subjects will be asked to give a trial blood samples.
- Orasure samples will be collected.

Ninth visit: 18 months (± 31 days) post 2nd vaccination

- Subjects will be asked to give a trial blood samples.
- Orasure samples will be collected.

Eleventh visit: 12 months (± 31 days) post 2nd vaccination

- Subjects will be asked to give a trial blood samples.
- Orasure samples will be collected.

Laboratory Analysis for the immunogenicity study

The trial blood samples will be collected at appropriate days (e.g. 0, 7, 14 and 21) after pH11N and seasonal vaccination (as detailed below) and at 6-24 months post vaccination to be used in serological assays and to study the kinetics of the T- and B-cellular responses. The serological assays (HI, SRH and neutralisation assays) will be carried out at the reference laboratories the National Institute of Biological Standards and Control and Health Protection Agency, UK and, University of Siena, Italy according to established standard operating procedures (SOP). The kinetics of the immune response elicited after vaccination with a H1N1 vaccine, including initial HI assays, will be conducted using blood and oral fluid samples at the University of Bergen. The detailed immunological response will be studied using a variety of immunological methods e.g. serum antibody (HI, protein array, ELISA for antibody class and IgG subclasses, neuraminidase assays), peripheral blood antibody secreting cells (e.g. ELISPOT and non secreted IgG from B

cells), lymphocyte profiles (e.g. Flow Cytometry for multifunctional T cells) and cytokine producing T-cells (e.g. ELISPOT, ELISA, multiplex analysis). The long lasting and cross reactive immunity will be studied (National Institute of Biological Standards and Control and Health Protection Agency, UK, University of Siena, Mount Sinai, University of Bergen) using established appropriate immunological assays e.g. ELISA, HI, SRH, or FACS analysis of lymphocytes stimulated *in vitro* with different H1 strains. Sera may also be used in other serological tests deemed appropriate for detection of influenza specific responses e.g. western blots, pseudotype assays and epitope mapping using the phage display system.

5. A description of the stopping rules for individuals, parts of trial and entire trial

The clinical investigator will evaluate the local and systemic adverse reactions of each subject. If a subject experiences serious unexpected local or systemic adverse reactions then this will be reported in PANVAK according to national guidelines from the Norwegian Institute for Public Health.

6. Maintenance of trial treatment codes and procedures for breaking the codes.

This study is an open study but the serological samples will be coded for laboratory analysis.

7. The identification of any data to be recorded directly in CRFs and to be considered to be source data

The study data will be verifiable to the source data, which necessitates access to all original recordings, such as subject list, blood sample log, vaccine administration log and adverse event forms. A daily log will be kept of all events related to the trial. On the day of vaccination, each subject in the immunogenicity study will be randomly allocated a unique subject identification number in order to protect confidentiality. The original source documentation will be a paper-based database, which will include the original hard copy of the results from the computer database (referred to below) of the serological results, and other information related to clinical trial. All databases will be secured against unauthorised access and confidentiality will be

maintained at all times. One paper database will be held at the immunisation clinic at the hospital and will contain personal data (name, sex, age, contact details, next of kin, and the National Personal Identification Number (personal number). The vaccination records will be stored in the National Institute for Public Health database (PANVAK) for pandemic vaccines with only the relevant Haukeland University Hospital staff having access to this database, by use of his/her personal access code to the hospital database. A paper database of adverse event forms will be kept at the Centre for Infection Control and these will be code with a unique number before the side reactions are entered into a computer database. The second database will be laboratory-based and contain the subject identification number and data relating to immunological responses, the only personal data will be age, sex and previous influenza history. The sponsor or her nominee will only have access to this database and it will be secured against unauthorised access.

6 Selection and Withdrawal of subjects

1. Subjects inclusion criteria

The following subjects will be considered eligible to enter the study or receive the second dose of vaccine if the following criteria are met:

Inclusion criteria

- Signed informed consent
- Subjects able to understand and comply with the study protocol and complete the Adverse Event Form
- Subjects able to attend the scheduled visits for the immunogenicity and kinetic study

2. Subjects exclusion criteria

The following subjects will not be considered eligible to enter the study:

- Persons with a history of anaphylaxis or serious reactions to any vaccine
- Person with known hypersensitivity to any of the vaccine components
- Persons who have had a temperature >38°C during the previous 72 hours
- Persons who have had an acute respiratory infection during the last 7 days
- Suspected non-compliance

3. Subjects withdrawal criteria

When and how to withdraw subjects from the trial

The clinical investigator will evaluate the local and systemic adverse reactions of each subject. If a subject experiences unexpected serious local or systemic reactions then these will be recorded in SYSVAK according to the national guidelines.

The type and timing of data to be collected for withdrawn subjects

No further trial samples will be collected from subjects who withdraw their informed consent for their participation in the study.

Whether and how subjects are to be replaced

Subjects will not be replaced if they are withdrawn or voluntarily withdraw from the trial.

The follow-up for subjects withdrawn from treatment

This study will gather information on the safety, tolerability and immunogenicity of the pandemic A/H1N1 vaccine administered as part of the mass vaccination campaign. As no treatment is given directly in this study there will be no follow up of subjects, however, subjects will be followed by Haukeland University Hospital when deemed clinically necessary.

7. Treatment of Subjects

1. Treatment to be administered including name of all products, the doses, the dosing schedule, the route of administration and the treatment periods including follow-up periods for the subjects for each product treatment group.

Subjects will receive one dose of pandemic A/H1N1 influenza vaccine by intramuscular injection into the deltoid muscle, as part of the mass vaccination campaign recommended by the Norwegian Institute for Public Health. Each subject will be enrolled in the study for 6 weeks for the adverse events and for 2 years for the immunogenicity study. They will be involved in the trial for a period of 6 weeks, during which blood samples will be collected at days 0, 7, 14 and 21 days after

pH11N and seasonal vaccination. All subjects will be asked to voluntarily provide trial blood samples at 3, 6, 12, 18 and 24 months after pH11N and seasonal vaccination to examine the longevity of the antibody response.

2. Medication permitted (including rescue medication) and not permitted before and /or during trial

All subjects enrolled in this trial may continue their normal medication throughout the study. Subjects may continue to take therapy for chronic medical conditions, but must be maintained on a stable regimen for at least 2 weeks prior to study entry as assessed by their Medical History.
If anaphylactic shock occurs after vaccination, it will be treated according to the standard routines at Haukeland University Hospital using hydrocortisone sodium succinate (Solu-Cortef 100mg), dexchlorfeniramine (5mg/mL), atropine (1mg/ml), adrenaline (1mg/mL and 0.1mg/mL). Use of other medication including over-the-counter products will be discouraged during the study. Investigational drugs are prohibited during the course of the study.

3. Procedures for monitoring subject compliance

This study is a vaccine study and forms part of the hospitals mass vaccination campaign. For subjects involved in the immunogenicity and kinetic studies, the daily log of events and CRF will be used to record if subjects have been vaccinated and provided the appropriate trial samples. All subjects vaccinated will be recorded in the PANVAK database according to the national guidelines.

8 Assessment of Efficacy

1. Specification of efficacy parameters

In the absence of true efficacy markers like prevention of illness and⁄or reduction of morbidity and mortality, serum anti-HA antibodies are the most commonly measured correlate of protection against influenza (65) or surrogates of efficacy. The CHMP have defined criteria for the annual update for seasonal influenza vaccines, which measure the serological responses by the haemagglutination inhibition or single radial haemolysis assays. An HI titre ≥ 40 (or an equivalent

SRH > 25mm^2) indicates 50% protective levels of serum antibody against influenza infection in man. The EU evaluation criteria in groups of at least 50 adults aged 18-60 approximately at 3 weeks after vaccination are:

- The number of seroconversions or significant increases in anti-haemagglutinin antibody titre should be > 40%;
- Mean geometric increase >2.5;
- The proportion of subjects achieving an HI titre ≥40 or SRH > 25mm^2 should be >70%

The CHMP require for marketing authorisation of a pandemic vaccine that the vaccine fulfils all current evaluation criteria for seasonal vaccines despite the immunological naivety of the host. The serological responses of vaccinees in the immunogenicity study will be evaluated and the ability of the vaccine to meet the CHMP criteria at different time points after vaccination will be determined.

In a pandemic situation, it is important to know how rapidly the immune response is elicited after vaccination with a novel subtype (60). Thus, the detailed kinetics of the immune response in immunologically naïve individuals will also be assessed through the induction of local and systemic antibody and cellular immune responses and results will be compared to published results on the response to current influenza vaccines in healthy adults. Furthermore, there is a clear need for improved understanding of the immune response elicited in human volunteers including the quality and repertoire of the antibody response which will be evaluated by ELISA and phage display, respectively. Two other important factors are the capacity of the vaccine to elicit long lasting immunity and cross reactive immunity to H1N1 influenza viruses (60). These will be evaluated by the standard serological assays and through the induction of local antibody and systemic cellular immune responses.

2. Methods and timing for assessing recording and analyzing efficacy parameters

In the immunogenicity study, blood samples will be taken for serological assays at days 0, 7, 14 and 21 after pH11N and seasonal vaccination. This study is an open study but all sera will be coded and blinded assays will be conducted by the University of Bergen, the National Institute of Biological Standards and Control, Health Protection Agency and University of Sienna, (SRH, HI, and neutralisation assays) on samples collected up to 21 ± 4 days after vaccination. When these

serological assays are completed the immunogenicity of the A/H1N1 influenza vaccine will then be assessed according to the three evaluation criteria defined by the CHMP and the following will be calculated:

- The number of subjects who seroconvert or show significant increases in anti-haemagglutinin antibody titre;
- The mean geometric increase;
- The proportion of subjects achieving an HI titre ≥40 or SRH > 25mm^2.

The immunogenicity of the vaccine will also evaluated by

- Producing a detailed time course of the immune response to vaccination including the kinetics of the T, B- and serum antibody responses
- The ability of the vaccine to induce long lasting immunity using the blood samples collected at 6,12, 18 and 24 months after pandemic and seasonal vaccination.
- The ability of the vaccine to induce cross-reactive immunity to other H1N1 strains.
- Examining the quality and repertoire of the antibody response.

Deviation from protocol

The investigators will adhere and try to avoid deviations from the protocol. The clinical investigators will document and explain any deviations from the approved protocol. Blood sampling for kinetic studies (T, B and antibody) can be collected within the following time frames after both the first and second doses of vaccine - day 0 ± 0, 7 ± 2, 14 ± 2, 21 ± 4. Subjects who do not adhere to these sampling time points have deviated from the protocol and their inclusion in the comparative serological analyses will be evaluated by the investigators.

Protocol amendments

The sponsor will treat any protocol amendments according to European guideline "Detailed guidance for the request for authorisation of a clinical trial on a medicinal product for human use to the competent authorities, notification of substantial amendments and declaration of the end of the trial". The regulatory authorities will be notified about substantial amendments and these amendments must be approved before being implemented.

9 Assessment of Safety

One of the main aims of this study is to evaluate the occurrence of adverse events in people vaccinated with the pandemic A/H1N1 vaccine. All subjects will be asked to fill in the adverse events form to record the incidence of local and systemic solicited side reactions. All adverse events and their follow up will be recorded in SYSVAK according to the national guidelines.

a. Adverse events (AE)

Note that the term vaccine refers to the pandemic A/H1N1 influenza vaccine in this study.

Definitions

Adverse events

An AE is any untoward medical occurrence in a patient or subject which does not necessarily have a causal relationship with a medical treatment. This includes any noxious, pathological or unintended change in anatomical, physiological, or metabolic functions as indicated by physical signs, symptoms and/or laboratory-detected changes. These might occur in any phase of the clinical study whether associated with or related to the vaccine or not. This includes also exacerbations of pre-existing conditions or events, intercurrent illnesses, or vaccine- or drug interactions. Anticipated day-to-day fluctuations of pre-existing conditions that do not represent a clinically significant exacerbation are not considered AEs. Discrete episodes of chronic conditions occurring during a study period should be reported as AE in order to assess changes in frequency or severity.

Serious adverse events (SAE)

SAEs are a subset of AEs. A SAE is defined as any untoward medical occurrence or effect at any dose that

- results in **death**
- is **life-threatening**: i.e. the subject was at risk of death at the time of the event; it does not refer to an event which hypothetically might have caused death if it were more severe
- results in persistent or significant **disability/incapacity**: i.e. results in a substantial disruption of the subject's ability to carry out normal life functions.
- requires in-patient **hospitalisation** or prolongation of existing hospitalisation: i.e. the subject is detained (usually at least an overnight stay) at the hospital or emergency ward for treatment that would not have been appropriate in the physician's office or in an out-patient setting. Hospitalisation for either elective surgery related to a pre-existing

condition which did not increase in severity or frequency following initiation of the study or for routine clinical procedures[1] (including hospitalisation for "social" reasons) that are not the result of an AE are **not** considered as SAEs

- is a **congenital anomaly or birth defect** in the offspring of a study subject
- is an **important medical event** that may jeopardise the subject or may require intervention to prevent one of the other outcomes listed above: e.g. interventions such as intensive treatment in an emergency room or at home for allergic bronchospasm; blood dyscrasias or convulsions that do not result in hospitalization, or development of drug dependency or drug abuse. Based on medical and scientific judgment, these events should usually be considered serious

Although **not** considered as SAE, cancer should be reported in the same way as SAEs.
Note: If anything untoward is reported during an elective procedure, that occurrence must be reported as an AE, either serious or non-serious according to the criteria defined above. When in doubt as to whether hospitalisation occurred or was necessary, the AE should be considered serious.

Surveillance, reporting, and documentation of adverse events

The recording of AEs is an essential part of study documentation. The clinical investigator is responsible for documenting all AEs as set out in the following sections.

Documentation of solicited adverse events

Solicited AEs are precisely defined events that the subjects are specifically asked about and which are documented by the subjects in the adverse event form. This form will be the source document.

The following local AEs at the vaccine injection site (injections site reactions) will be documented in the Adverse Event Form:

- Pain
- Erythema
- Ecchymosis
- Induration
- Itching
- Swelling

---

[1] A procedure which may take place during the study period and should not interfere with the vaccine administration or any of the ongoing protocol-specific procedures.

The following solicited systemic AEs will be documented in the adverse event form and transcribed by the investigator into the CRF:

- Body temperature (oral) ≥ 38°C
- Malaise
- Shivering
- Fatigue
- Headache
- Sweating
- Myalgia
- Arthralgia
- Diarrhoea
- Respiratory symptoms

An Adverse Event Form will be issued to all subjects instructing them to record their daily local and systemic adverse events for as long as symptoms persist. The subjects will provide information on the presence or absence of solicited local and systemic adverse events. The use of antipyretic/relief medication and any changes in medication will also be recorded. Subjects will score the severity of symptoms as follows:

Blank – no adverse symptoms

1 -symptom occurred but not severe enough to cause inconvenience

2 -symptom occurred severe enough to interfere with daily activities but required minimal or no medical intervention

3-symptom occurred severe enough to markedly interfere with daily activities; required medical consultation

At each visit for the immunogenicity study, all AEs, either observed by the clinical investigator or his nominee or reported by the subjects spontaneously or in response to a direct question will be evaluated by the investigator.

As a consistent method to find out about adverse events, the investigator should use a non-leading question, such as:

"Have you felt different since receiving the vaccine or since the previous visit?"

The subjects will be instructed to contact the clinical investigators immediately should they experience any signs or symptoms they perceive as serious during the period extending from the first study-specific procedure up to and including 6 months (minimum 180 days) after the last administration of the vaccine.

Documentation of adverse events

For the immunogenicity study, any AE occurring within 3 weeks (minimum 17 days) following vaccination will be recorded on the AE Page of the CRF, irrespective of its severity or potential relationship to the vaccine.

Any SAE brought to the attention of the investigator from study start up to 6 months (at least 180 days) after vaccination of that subject will be recorded. AEs not previously documented in the adverse event form will be recorded in the AE page of the CRF. AEs should be documented in terms of signs and symptoms observed by the investigator or reported by subjects. Whenever possible, a medical diagnosis should be made. The nature of each event, date and (where appropriate) time of onset, outcome, severity and causal relationship should be established. Details of any symptomatic/corrective treatment should be recorded on the appropriate page of the CRF.

Hospitalisation for routine clinical procedures (including hospitalisation for "social" reasons) that are not the result of an AE are not considered AEs but will be recorded on the AE page of the CRF. The same applies for hospitalisation for elective procedures related to a pre-existing condition that did not increase in severity or frequency during the study. If hospitalisation was planned before first administration of the vaccine, it will be documented in the Medical History Page of the CRF (see below).

The following events will be documented in the Medical History Page of the CRF:

- AEs which occur after informed consent was obtained, but before the first vaccine administration
- Pre-existing conditions or signs and/or symptoms present in a subject before study start. This includes conditions which were not recognized at study entry but later during the study period
- Hospitalisation arising from a pre-existing condition and planned before the first administration of the vaccine

Reporting of serious adverse events

All SAEs will be reported immediately by the investigator without filtration into SYSVAK according to national guidelines, independent of their association or causal relation to the vaccine. The initial notification should include all known information regarding the SAE (which may be

minimal, but should include sufficient information to permit identification of the reporter, the subject, the vaccine the SAE, and the date of onset of the SAE). The initial report should then be followed by submission of a completed SAE report to SYSVAK as soon as possible but at latest within the time frame specified in the national guidelines.
The completed SAE Report Form should detail all relevant aspects of the SAE, all actions taken by the investigator, the causality, and the outcome of the event. Any new information obtained regarding the SAE must be reported immediately. The SAE Report Forms should be used for documentation of the SAE, any actions taken, the outcome, and for follow-up reports. When applicable, hospital case records and autopsy reports should be obtained by the investigator and forwarded to the Study Contact for SAE Reporting.
The investigator must report SAEs to the appropriate Ethics Committee, if requested by the committee, and/or according to local legal requirements.

The reporting obligations for SUSARs will be fulfilled as required by the applicable law.

Causality of adverse events

Every effort should be made by the investigator to explain any AE and assess its potential causal relationship to administration of the vaccine. This applies to all AEs, i.e. to both non-serious and serious adverse events.
To which degree of certainty an AE can be attributed to administration of the vaccine or to alternative causes (e.g. natural history of the underlying diseases, concomitant therapy) will be determined by how well the event can be understood in terms of one or more of the following:

- A reaction of a similar nature has been previously observed with this type of medicinal product and/or formulation
- The event has often been reported in literature for similar types of medicinal products
- The event has been temporally associated with vaccine administration or reproduced on re-administration of the vaccine

By definition, all solicited local AEs occurring at the application site (i.e. all solicited local reactions) will be considered related to the vaccine administration (injection site reactions).

Causality of all other AEs should be assessed by the investigator based on the following:
In your opinion, is there a reasonable possibility that the AE was caused by the vaccine?

Related	there is suspicion that there is a relationship between vaccine and AE (without determining the extent of probability); there is a reasonable possibility that the vaccine contributed to the AE
Unrelated	there is no suspicion that there is a relationship between vaccine and AE; there are other more likely causes and administration of the vaccine is not suspected to have contributed to the AE

Severity of adverse events

The severity of unsolicited AEs will be graded on a 3-point scale as follows:

**Grade**	**Definition**
1 (mild)	Discomfort noted, but no disruption of normal daily activity; slightly bothersome; relieved with or without symptomatic treatment.
2 (moderate)	Discomfort sufficient to reduce or affect normal daily activity to some degree; bothersome; interferes with normal daily activities; only partially relieved with symptomatic treatment.
3 (severe)	Discomfort sufficient to reduce or affect normal daily activity considerably; prevents regular activities; not relieved with symptomatic treatment.

Follow-up of ongoing adverse events and assessment of outcome

Follow-up of non-serious adverse events for subjects in the immunogenicity study.
Non-serious AEs already documented in the CRF at a previous assessment and designated as ‘ongoing’ should be reviewed at subsequent visits. If the event has resolved, the documentation in the CRF should be completed. If the frequency or severity of a non-serious AE changes significantly, a new record of the AE has to be started. If the AE becomes serious, the procedures for reporting of SAEs have to be followed (see above).
Ongoing non-serious AEs will be followed until the end of the active study phase.

Outcome will be assessed as:

1 Resolved (no sequelae)

2 Resolved (with sequelae)

3 Ongoing

4 Death

5 Unknown

Follow-up of serious adverse events

All SAEs must be followed-up according to national guidelines until the event has either resolved, subsided, stabilized, disappeared, or is otherwise explained, or the study subject is lost to follow-up, but no longer than 6 months after the last vaccination.

All follow-up activities must be reported in a timely manner to SYSVAK. All fields of the form with additional or changed information must be completed and the SAE Report Form should be forwarded to the Study Contact for SAE Reporting as soon as possible but at the latest within 7 calendar days after receipt of the new information. Reports related to the subsequent course of any SAE reported for any subject will be submitted according to National guidelines.

Treatment of adverse events

Treatment of any AE is at the sole discretion of the investigator and according to current available best treatment. The applied measures should be recorded in the CRF.

10. Statistics

1. A description of the statistical methods to be employed

The main aim of this study is to evaluate the tolerability and immunogenicity of the pandemic H1N1 vaccine. Adverse reactions will be collected from up to 8000 volunteers and will be analysed by the Fisher's exact and Mantel-Haenszel tests. In the immunogenicity study, immunological analyses will be available form up to 550 volunteers. The number of responders with detectable antibody was compared by the Mantel-Haenszel test using the Breslow-Day test

analysis for the homogeneity of the odd ratios. The paired two-sided Student's t-test will be used to analyse the antibody and cellular responses.

2. The number of subjects planned to be enrolled and reason for choice of sample size

All subjects included for mass vaccination will be included in the adverse events analysis which should provide a database containing a maximum of 8000 reports. The CHMP criteria for licensure of seasonal vaccines require data from 50 volunteers and this number ahs been chosen as the subset of volunteers to be included in the kinetic study. Up to 500 subjects willing to provide three blood samples will be included in the immunogenicity studies.

<u>Deviation from protocol and procedures for accounting for missing data</u>

The investigators will adhere and try to avoid deviations from the protocol. All trial blood samples can be collected within the following time frames after vaccination; Day 0 $\pm$ 0, 7 $\pm$ 2, 14 $\pm$ 2, 21 $\pm$ 4. The clinical investigators will document and explain any deviations from the approved protocol. Subjects who do not adhere to these sampling time points have deviated from the protocol and their inclusion in the comparative analyses will be evaluated by the investigators.

11. Direct access to source data/documentation

The study data will be verifiable to the source data, which necessitates access to all original recordings, such as vaccination records, informed consent, subject list, serum sample log, vaccine administration log and Adverse Event Form. A daily log will be kept of all events related to the immunogenicity study. The clinical investigators are allowed access to the subject's hospital medical records. The subjects will be informed of this and will be signing their agreement when giving informed consent.

On the day of vaccination each subject in the immunogenicity study will be randomly allocated a unique subject identification number in order to protect confidentiality. Two computer databases will be used. All original vaccination records will be stored in Sysvak as defined by national guidelines and patient confidentiality will be maintained according to the national established guidelines. The second database will be laboratory-based and the only personal data will be age and sex, previous influenza history and data relating to immunological responses. This database will only be accessed by the Principal Investigator or her nominee and the entry point will only be

identified by the subject identification number. However, two paper-based databases (one clinical database and one laboratory based database) will be established and they will be considered as the original source documentation. The paper-based databases will include, the original hard copy of the documents from computer databases (e.g. serological results), adverse events forms, CRFs and other information related to clinical trial. One database will be held at the immunisation clinic at the hospital and will contain personal data (name, sex, age, contact details, next of kin, and the National Personal Identification Number (Personal number). All databases will be secured against unauthorised access and confidentiality will be maintained at all times. Monitors, Auditors, Inspectors, and Regulatory Authorities will have access to the paper database if required.

12 Quality Control and Quality Assurance

All the data will be generated, recorded and reported under restricted quality assured system in accordance with GCP requirements. The principle investigator will assign a qualified person to double check and quality control (according to established SOPs) all data entries to ensure that they are correct and complete. Any changes, corrections or amendments in the original data and records will be signed and dated by the authorized person. Furthermore, the changes will be conducted in a proper way to guarantee the traceability of the original data. All the source data, reports and documents will be accessible for internal auditing and to inspection by regulatory authorities.

13. Ethics

The pandemic A/H1N1 vaccine will be used to provide protection against pandemic influenza in millions of people. As font line health care workers are a prioritized group it is important to compile a large database to assess the incidence of adverse reactions. The current safety database for the mock up vaccine has been considered sufficient by the CHMP to describe adverse reactions that occur uncommonly and to give an indication of any rare events. However, some adverse reactions may occur very rarely after influenza vaccination. We cannot predict if higher rates might be observed with Pandemrix for pandemic H1N1 virus compared to seasonal influenza vaccines, but is it vitally important to have data available from a large cohort of vaccinees.

Good Clinical Practice

The study will be conducted according to the Declaration of Helsinki, ICH / Guidelines on Good Clinical Practice for Trials on Medicinal Products in the European Community and local legal requirements.

Regulatory authority approval

The pandemic vaccine will be a medicinal product licensed by the Norwegian Medicines Agency.

Ethics committee approval

The principal investigator will submit the protocol, subject information, informed consent, and other study-related documents as required by applicable laws and regulations for approval to the relevant independent ethics committee (Regional komité for medisinsk forskningsetikk, Vest (REK Vest)) before study commencement. The ethics committee will be informed of all subsequent protocol amendments and of SUSARs occurring during the trial. The trial will not commence until approved by all relevant authorities.

Patient informed consent

Prior to entry, the investigators will inform the volunteers of the purpose, nature, possible benefits and potential hazards of the study including any discomfort or adverse event which may occur. Subjects will be given the necessary time to ask for further information or clarification. The subject will then be given suitable time to decide if he or she wishes to participate in the trial. The subject can at any time point withdraw his/her consent, without detriment. No financial incentives will be offered. Each subject will provide a signed informed consent prior to any study-related activities.

Removal/withdrawal of subjects from treatment or assessment

Subjects may at any time withdraw their informed consent for their participation in the study without any resulting detriment. If a subject withdraws from this trial he/she will not be replaced. The clinical investigator may also withdraw a subject if it is believed to be medically in the best interest of the subject or if the subject cannot comply with the protocol.

14 Data handling and record keeping

The clinical investigators will be responsible that data reported in the CRF and the adverse event forms are complete, accurate and legible record for each subject. The clinical investigators will also ensure that all data reported on the CRF is consistent with the original hospital records and that any corrections are appropriately dated and initialled. The clinical investigators will provide adequate instructions on completion of the adverse event form to each subject.

Storage and retention of study documentation

The documentation for this study will be stored as hard copy documents in a secure office where only the sponsor or his nominee has access to as required by GCP guidelines. The documents will be retained for a minimum of 15 years.

15 Financing and Insurance

The sponsor of this trial is the University of Bergen and Haukeland University Hospital. The trial will be funded by the Influenza Centre, University of Bergen and Helse Vest.
If a subject is harmed by his/her participation in this clinical trial, he/she will be compensated according to established guidelines. Liability for pandemic vaccination rests nationally and the pandemic vaccine will be licensed by EMEA. The investigators will purchase a drug liability insurance policy with the Drug Liability Association (Legemiddelansvarsforeningen) which will insure all subjects in this trial according to Produktansvarsloven 23rd December 1988 nr. 104 chapter 3.

16 Publication Policy

The results of this study will be published in international peer reviewed journals. A substantial amount of information will be generated by the study. Publications arising out of the work will focus on:

- The safety and comparative reactogenicity of pandemic H1N1 vaccines in an immunologically naïve population.
- The kinetics of the antibody and B and T cellular responses
- Evaluation of the ability of adjuvanted pandemic H1N1 influenza vaccine to fulfil the CHMP criteria
- Detailed time course of the immune response to vaccination with a novel H1N1 influenza subtype including the kinetics and quality of the serum antibody and T and B cellular responses and epitope mapping
- The longevity of the immunity induced by the vaccine
- The ability of the vaccine to elicit cross reactive immunity against other H1 strains.

17 Supplements

18 References

1. World Health Organization (WHO). World now at the start of 2009 influenza pandemic. 2009 [cited 2009 11 June]; Available from: http://www.who.int/mediacentre/news/statements/2009/h1n1_pandemic_phase6_20090611/en/index.html.
2. Zimmer SM, Burke DS. Historical perspective--Emergence of influenza A (H1N1) viruses. N Engl J Med. 2009 Jul 16;361(3):279-85.
3. Fraser C, Donnelly CA, Cauchemez S, Hanage WP, Van Kerkhove MD, Hollingsworth TD, et al. Pandemic potential of a strain of influenza A (H1N1): early findings. Science. 2009 Jun 19;324(5934):1557-61.
4. Smith GJ, Vijaykrishna D, Bahl J, Lycett SJ, Worobey M, Pybus OG, et al. Origins and evolutionary genomics of the 2009 swine-origin H1N1 influenza A epidemic. Nature. 2009 Jun 25;459(7250):1122-5.
5. Neumann G, Noda T, Kawaoka Y. Emergence and pandemic potential of swine-origin H1N1 influenza virus. Nature. 2009 Jun 18;459(7249):931-9.
6. Garten RJ, Davis CT, Russell CA, Shu B, Lindstrom S, Balish A, et al. Antigenic and genetic characteristics of swine-origin 2009 A(H1N1) influenza viruses circulating in humans. Science. 2009 Jul 10;325(5937):197-201.
7. Serum cross-reactive antibody response to a novel influenza A (H1N1) virus after vaccination with seasonal influenza vaccine. MMWR Morb Mortal Wkly Rep. 2009 May 22;58(19):521-4.
8. Cox RJ, Brokstad KA, Ogra P. Influenza virus: immunity and vaccination strategies. Comparison of the immune response to inactivated and live, attenuated influenza vaccines. Scand J Immunol. 2004 Jan;59(1):1-15.
9. Wood JM, Robertson JS. Reference viruses for seasonal and pandemic influenza vaccine preparation. Influenza and Other Respiratory Viruses. 2007;1:5-9.
10. Treanor JJ, Campbell JD, Zangwill KM, Rowe T, Wolff M. Safety and immunogenicity of an inactivated subvirion influenza A (H5N1) vaccine. N Engl J Med. 2006 Mar 30;354(13):1343-51.
11. Treanor JJ, Wilkinson BE, Masseoud F, Hu-Primmer J, Battaglia R, O'Brien D, et al. Safety and immunogenicity of a recombinant hemagglutinin vaccine for H5 influenza in humans. Vaccine. 2001 Feb 8;19(13-14):1732-7.
12. Stephenson I, Nicholson KG, Wood JM, Zambon MC, Katz JM. Confronting the avian influenza threat: vaccine development for a potential pandemic. Lancet Infect Dis. 2004 Aug;4(8):499-509.
13. Haaheim LR. Vaccines for an influenza pandemic: scientific and political challenges. Influenza and Other Respiratory Viruses. 2007;1(2):55-60.
14. Keitel WA, Atmar RL. Preparing for a possible pandemic: influenza A/H5N1 vaccine development. Curr Opin Pharmacol. 2007 Oct;7(5):484-90.
15. Palache AM, vd Velden JW. Influenza vaccination in asthma. Lancet. 1992 Mar 21;339(8795):741.
16. Beyer WE, Palache AM, de Jong JC, Osterhaus AD. Cold-adapted live influenza vaccine versus inactivated vaccine: systemic vaccine reactions, local and systemic antibody response, and vaccine efficacy. A meta-analysis. Vaccine. 2002 Jan 31;20(9-10):1340-53.
17. Nichol KL. Efficacy/clinical effectiveness of inactivated influenza virus vaccines in adults. In: Nicholson KG, Webster RG, Hay AJ, editors. Textbook of influenza. first ed. Oxford: Blackwell science; 1998. p. 358-72.
18. vaksinasjon Vo. Folkehelse. 2007.

19. Lasky T, Terracciano GJ, Magder L, Koski CL, Ballesteros M, Nash D, et al. The Guillain-Barre syndrome and the 1992-1993 and 1993-1994 influenza vaccines. N Engl J Med. 1998 Dec 17;339(25):1797-802.
20. Askonas BA, Lin YL. An influenza specific T-killer clone is restricted to H-2Ld and cross-reacts with Dk region. Immunogenetics. 1982;16(1):83-7.
21. Cox RJ, Brokstad KA, Zuckerman MA, Wood JM, Haaheim LR, Oxford JS. An early humoral immune response in peripheral blood following parenteral inactivated influenza vaccination. Vaccine. 1994 Aug;12(11):993-9.
22. Powers DC, Smith GE, Anderson EL, Kennedy DJ, Hackett CS, Wilkinson BE, et al. Influenza A virus vaccines containing purified recombinant H3 hemagglutinin are well tolerated and induce protective immune responses in healthy adults. J Infect Dis. 1995 Jun;171(6):1595-9.
23. Brokstad KA, Cox RJ, Olofsson J, Jonsson R, Haaheim LR. Parenteral influenza vaccination induces a rapid systemic and local immune response. J Infect Dis. 1995 Jan;171(1):198-203.
24. Brokstad KA, Cox RJ, Oxford JS, Haaheim LR. IgA, IgA subclasses, and secretory component levels in oral fluid collected from subjects after parental influenza vaccination. J Infect Dis. 1995 Apr;171(4):1072-4.
25. El-Madhun AS, Cox RJ, Haaheim LR. The effect of age and natural priming on the IgG and IgA subclass responses after parenteral influenza vaccination. J Infect Dis. 1999 Oct;180(4):1356-60.
26. el-Madhun AS, Cox RJ, Soreide A, Olofsson J, Haaheim LR. Systemic and mucosal immune responses in young children and adults after parenteral influenza vaccination. J Infect Dis. 1998 Oct;178(4):933-9.
27. Janeway CA TP, Walport M et al. . Immunobiology2005.
28. Russell JH, Ley TJ. Lymphocyte-mediated cytotoxicity. Annu Rev Immunol. 2002;20:323-70.
29. Cox RJ, Madhun AS, Hauge S, Sjursen H, Major D, Kuhne M, et al. A phase I clinical trial of a PER.C6 cell grown influenza H7 virus vaccine. Vaccine. 2009 Mar 18;27(13):1889-97.
30. Lipatov AS, Hoffmann E, Salomon R, Yen HL, Webster RG. Cross-protectiveness and immunogenicity of influenza A/Duck/Singapore/3/97(H5) vaccines against infection with A/Vietnam/1203/04(H5N1) virus in ferrets. J Infect Dis. 2006 Oct 15;194(8):1040-3.
31. Cox RJ DM, Solveig Hauge , Abdullah S. Madhun, Karl A. Brokstad, Mirjam Kuhne, Jon Smith, Frederick R. Vogel, Maria Zambon, Lars R. Haaheim, John Wood. A cell-based H7N1 split influenza virion vaccine confers protection in mouse and ferret challenge models. Influenza and Other Respiratory Viruses. 2009;3(3):107-17.
32. O'Neill E, Krauss SL, Riberdy JM, Webster RG, Woodland DL. Heterologous protection against lethal A/HongKong/156/97 (H5N1) influenza virus infection in C57BL/6 mice. J Gen Virol. 2000 Nov;81(Pt 11):2689-96.
33. Droebner K, Haasbach E, Fuchs C, Weinzierl AO, Stevanovic S, Buttner M, et al. Antibodies and CD4(+) T-cells mediate cross-protection against H5N1 influenza virus infection in mice after vaccination with a low pathogenic H5N2 strain. Vaccine. 2008 Dec 9;26(52):6965-74.
34. Burgers WA, Chege GK, Muller TL, van Harmelen JH, Khoury G, Shephard EG, et al. Broad, high-magnitude and multifunctional CD4+ and CD8+ T-cell responses elicited by a DNA and modified vaccinia Ankara vaccine containing human immunodeficiency virus type 1 subtype C genes in baboons. J Gen Virol. 2009 Feb;90(Pt 2):468-80.
35. Seder RA, Darrah PA, Roederer M. T-cell quality in memory and protection: implications for vaccine design. Nat Rev Immunol. 2008 Apr;8(4):247-58.
36. Galli G, Medini D, Borgogni E, Zedda L, Bardelli M, Malzone C, et al. Adjuvanted H5N1 vaccine induces early CD4+ T cell response that predicts long-term persistence of protective antibody levels. Proc Natl Acad Sci U S A. 2009 Mar 10;106(10):3877-82.

37. Wrammert J, Ahmed R. Maintenance of serological memory. Biol Chem. 2008 Mar 6.
38. Crotty S, Aubert RD, Glidewell J, Ahmed R. Tracking human antigen-specific memory B cells: a sensitive and generalized ELISPOT system. J Immunol Methods. 2004 Mar;286(1-2):111-22.
39. Drane DL, Osato SS. Using the neurobehavioral cognitive status examination as a screening measure for older adults. Arch Clin Neuropsychol. 1997;12(2):139-43.
40. Hobson D, Curry RL, Beare AS, Ward-Gardner A. The role of serum haemagglutination-inhibiting antibody in protection against challenge infection with influenza A2 and B viruses. J Hyg (Lond). 1972 Dec;70(4):767-77.
41. Schild GC, Pereira MS, Chakraverty P. Single-radial-hemolysis: a new method for the assay of antibody to influenza haemagglutinin. Applications for diagnosis and seroepidemiologic surveillance of influenza. . Bull World Health Organ. 1975;52(1): 43-50.
42. WHO. WHO strategic action plan for pandemic influenza 2007.
43. Committee. Guideline On Dossier Structure And Content Of Marketing Authorisation Applications For Influenza Vaccines Derived From Strains With A Pandemic Potential For Use Outside Of The Core Dossier Context EMEA/CHMP/VWP/263499/2006. European Agency for the Evaluation of Medicinal Products, July 24, 2006. 1996.
44. Hehme N, Engelmann H, Kunzel W, Neumeier E, Sanger R. Pandemic preparedness: lessons learnt from H2N2 and H9N2 candidate vaccines. Med Microbiol Immunol (Berl). 2002 Dec;191(3-4):203-8.
45. Stephenson I, Nicholson KG, Colegate A, Podda A, Wood J, Ypma E, et al. Boosting immunity to influenza H5N1 with MF59-adjuvanted H5N3 A/Duck/Singapore/97 vaccine in a primed human population. Vaccine. 2003 Apr 2;21(15):1687-93.
46. Stephenson I, Nicholson KG, Gluck R, Mischler R, Newman RW, Palache AM, et al. Safety and antigenicity of whole virus and subunit influenza A/Hong Kong/1073/99 (H9N2) vaccine in healthy adults: phase I randomised trial. Lancet. 2003 Dec 13;362(9400):1959-66.
47. Lin J, Zhang J, Dong X, Fang H, Chen J, Su N, et al. Safety and immunogenicity of an inactivated adjuvanted whole-virion influenza A (H5N1) vaccine: a phase I randomised controlled trial. Lancet. 2006 Sep 16;368(9540):991-7.
48. Leroux-Roels I, Borkowski A, Vanwolleghem T, Drame M, Clement F, Hons E, et al. Antigen sparing and cross-reactive immunity with an adjuvanted rH5N1 prototype pandemic influenza vaccine: a randomised controlled trial. Lancet. 2007 Aug 18;370(9587):580-9.
49. Ehrlich HJ, Muller M, Oh HM, Tambyah PA, Joukhadar C, Montomoli E, et al. A clinical trial of a whole-virus H5N1 vaccine derived from cell culture. N Engl J Med. 2008 Jun 12;358(24):2573-84.
50. Bresson JL, Perronne C, Launay O, Gerdil C, Saville M, Wood J, et al. Safety and immunogenicity of an inactivated split-virion influenza A/Vietnam/1194/2004 (H5N1) vaccine: phase I randomised trial. Lancet. 2006 May 20;367(9523):1657-64.
51. Hehme N, Engelmann H, Kuenzel W, Neumeier E, Saenger R. Immunogenicity of a monovalent, aluminum-adjuvanted influenza whole virus vaccine for pandemic use. Virus Res. 2004 Jul;103(1-2):163-71.
52. Keitel WA, Campbell JD, Treanor JJ, Walter EB, Patel SM, He F, et al. Safety and Immunogenicity of an Inactivated Influenza A/H5N1 Vaccine Given with or without Aluminum Hydroxide to Healthy Adults: Results of a Phase I-II Randomized Clinical Trial. J Infect Dis. 2008 Nov 1;198(9):1309-16.
53. Bernstein DI, Edwards KM, Dekker CL, Belshe R, Talbot HK, Graham IL, et al. Effects of adjuvants on the safety and immunogenicity of an avian influenza H5N1 vaccine in adults. J Infect Dis. 2008 Mar 1;197(5):667-75.
54. Nicholson KG, Colegate AE, Podda A, Stephenson I, Wood J, Ypma E, et al. Safety and antigenicity of non-adjuvanted and MF59-adjuvanted influenza A/Duck/Singapore/97 (H5N3)

vaccine: a randomised trial of two potential vaccines against H5N1 influenza. Lancet. 2001 Jun 16;357(9272):1937-43.
55. Stephenson I, Bugarini R, Nicholson KG, Podda A, Wood JM, Zambon MC, et al. Cross-reactivity to highly pathogenic avian influenza H5N1 viruses after vaccination with nonadjuvanted and MF59-adjuvanted influenza A/Duck/Singapore/97 (H5N3) vaccine: a potential priming strategy. J Infect Dis. 2005 Apr 15;191(8):1210-5.
56. (IVR) WHOIfVR. Tables on the Clinical trials of pandemic influenza prototype vaccines. 2008.
57. Ninomiya A, Imai M, Tashiro M, Odagiri T. Inactivated influenza H5N1 whole-virus vaccine with aluminum adjuvant induces homologous and heterologous protective immunities against lethal challenge with highly pathogenic H5N1 avian influenza viruses in a mouse model. Vaccine. 2007 May 4;25(18):3554-60.
58. Suguitan AL, Jr., McAuliffe J, Mills KL, Jin H, Duke G, Lu B, et al. Live, attenuated influenza A H5N1 candidate vaccines provide broad cross-protection in mice and ferrets. PLoS Med. 2006 Sep;3(9):e360.
59. Mahmood K, Bright RA, Mytle N, Carter DM, Crevar CJ, Achenbach JE, et al. H5N1 VLP vaccine induced protection in ferrets against lethal challenge with highly pathogenic H5N1 influenza viruses. Vaccine. 2008 Oct 3;26(42):5393-9.
60. CHMP. Core SPC for pandemic influenza vaccines. EMEA/CHMP/VEG/193031/2004 2004.
61. Madhun AS, Akselsen PE, Sjursen H, Pedersen G, Svindland S, Nostbakken JK, et al. An adjuvanted pandemic influenza H1N1 vaccine provides early and long term protection in health care workers. Vaccine. 2010 Dec 16;29(2):266-73.
62. Zuckerman M, Cox R, Taylor J, Wood J, Haaheim L, Oxford J. Rapid immune response to influenza vaccination. Lancet. 1993 Oct 30;342(8879):1113.
63. Brokstad KA, Cox RJ, Major D, Wood JM, Haaheim LR. Cross-reaction but no avidity change of the serum antibody response after influenza vaccination. Vaccine. 1995 Nov;13(16):1522-8.
64. el-Madhun AS, Cox RJ, Seime A, Sovik O, Haaheim LR. Systemic and local immune responses after parenteral influenza vaccination in juvenile diabetic patients and healthy controls: results from a pilot study. Vaccine. 1998 Jan-Feb;16(2-3):156-60.
65. Cox NJ, Subbarao K. Influenza. Lancet. 1999 Oct 9;354(9186):1277-82.
